# Freeze-dried *Nannochloropsis oceanica* biomass protects eicosapentaenoic acid (EPA) from metabolization in the rumen of lambs

**DOI:** 10.1038/s41598-021-01255-w

**Published:** 2021-11-08

**Authors:** Ana C. M. Vítor, Alexandra E. Francisco, Joana Silva, Mário Pinho, Sharon A. Huws, José Santos-Silva, Rui J. B. Bessa, Susana P. Alves

**Affiliations:** 1 Faculdade de Medicina Veterinária, Universidade de Lisboa, Av. da Universidade Técnica, 1300-477 Lisboa, Portugal; 2 CIISA - Centro de Investigação Interdisciplinar em Sanidade Animal, Avenida da Universidade Técnica, 1300-477 Lisboa, Portugal; 3 Polo de Investigação de Santarém, Instituto Nacional de Investigação Agrária E Veterinária (INIAV-Santarém), 2005-048 Vale de Santarém, Portugal; 4 Allmicroalgae, Rua 25 Abril, 2445-413 Pataias, Portugal; 5 School of Biological Sciences, Institute for Global Food Security, Queen’s University Belfast, Belfast, UK

**Keywords:** Fatty acids, Animal physiology

## Abstract

Eicosapentaenoic acid (EPA) from freeze-dried biomass of *Nannochloropsis oceanica* microalgae resists ruminal biohydrogenation in vitro, but in vivo demonstration is needed. Therefore, the present study was designed to test the rumen protective effects of *N. oceanica* in lambs. Twenty-eight lambs were assigned to one of four diets: Control (C); and C diets supplemented with: 1.2% *Nannochloropsis* sp. oil (O); 12.3% spray-dried *N. oceanica* (SD); or 9.2% *N. oceanica* (FD), to achieve 3 g EPA /kg dry matter. Lambs were slaughtered after 3 weeks and digestive contents and ruminal wall samples were collected. EPA concentration in the rumen of lambs fed FD was about 50% higher than lambs fed SD or O diets. Nevertheless, the high levels of EPA in cecum and faeces of animals fed *N. oceanica* biomass, independently of the drying method, suggests that EPA was not completely released and absorbed in the small intestine. Furthermore, supplementation with EPA sources also affected the ruminal biohydrogenation of C18 fatty acids, mitigating the shift from the *t*10 biohydrogenation pathways to the *t*11 pathways compared to the Control diet. Overall, our results demonstrate that FD *N. oceanica* biomass is a natural rumen-protected source of EPA to ruminants.

## Introduction

The health benefits associated with the consumption of omega-3 long-chain polyunsaturated fatty acids (n-3 LC-PUFA), particularly eicosapentaenoic acid (EPA, 20:5n-3) and docosahexaenoic acid (DHA, 22:6n-3) are well known^1, 2^. However, in ruminant edible fats their content is very low even when animals are supplemented with enriched n-3 LC-PUFA diets. The explanation for this finding relies on the ruminal microbiota intervention over the dietary lipids. Indeed, rumen microbes have the capacity to hydrolyse lipids and subsequently biohydrogenate the unsaturated fatty acids (FA). The biohydrogenation involves isomerization and hydrogenation of FA double bonds, forming a wide range of FA intermediates and saturated FA as end products, which will further alter the FA profile of ruminant edible products^3^.

Strategies to enhance the content of n-3 LC-PUFA in ruminant edible fat include the dietary supplementation with products derived from the marine food chain, as fish oil or microalgae, which are naturally enriched in EPA and DHA^4, 5^. However, the efficiency is rather low as it is well established that both EPA and DHA undergo extensive biohydrogenation in the rumen^3–5^. Thus, rumen-protected marine-derived supplements could be the most effective way to increase the concentration of EPA and DHA in ruminant derived foods.

Indeed, several rumen protection technologies have been proposed but often with low efficacy and with application difficulties^6^. Furthermore, the bypass must allow post-ruminal release once it reaches the small intestine. Calcium salts have a disadvantage related to dissociation in lower pH, the limited amount of protectable polyunsaturated FA (PUFA) and the need of free FA to create an ionic bond between the free carboxyl group of the FA and Ca ions. Formaldehyde is a carcinogenic compound; the reaction is untargeted, and it is an expensive resource. Other methods such as fatty acyl amides, non-enzymatic browning, lipid composite gels, encapsulation within lipids and protein crosslinking, all have several disadvantages as many of these technologies use harmful products, are not cost-effective, or are lacking consistency regarding rumen protection efficiency^6^. Thus, the identification of PUFA sources naturally protected from rumen metabolism is highly relevant and promising.

Consequently, using in vitro batch incubations with rumen inoculum, our team was able to identify that biomass of *Nannochloropsis oceanica* microalgae was a partially protected source of EPA^7^. The protection against rumen biohydrogenation was moderate with spray-dried (SD) biomass and exceptionally high when freeze-dried (FD) biomass was used. Microalgae are usually included in diet formulations after dehydration of the slurry biomass using industrial-scale SD technology. Dehydration by freeze-drying seems better in preserving the structural components and nutritional properties than SD^8, 9^. Thus, the higher rumen-protection of EPA observed in vitro with FD biomass was probably due to better preservation of *N. oceanica* cell walls than with SD biomass. However, the better preservation of cell walls might limit the post-ruminal release and availability for the EPA's absorption.

The objective of the present study was to evaluate FD *N. oceanica* as a natural rumen-protected source of EPA in vivo. We hypothesized that lambs fed a diet containing FD *N. oceanica* biomass would present a higher content of EPA through the main gastrointestinal tract compartments, comparing to those fed diets with SD biomass, or with *Nannochloropsis sp*. Oil (O).

## Results

### Abundance of EPA in digestive tract and whole tract apparent digestibility

**Figure 1:**
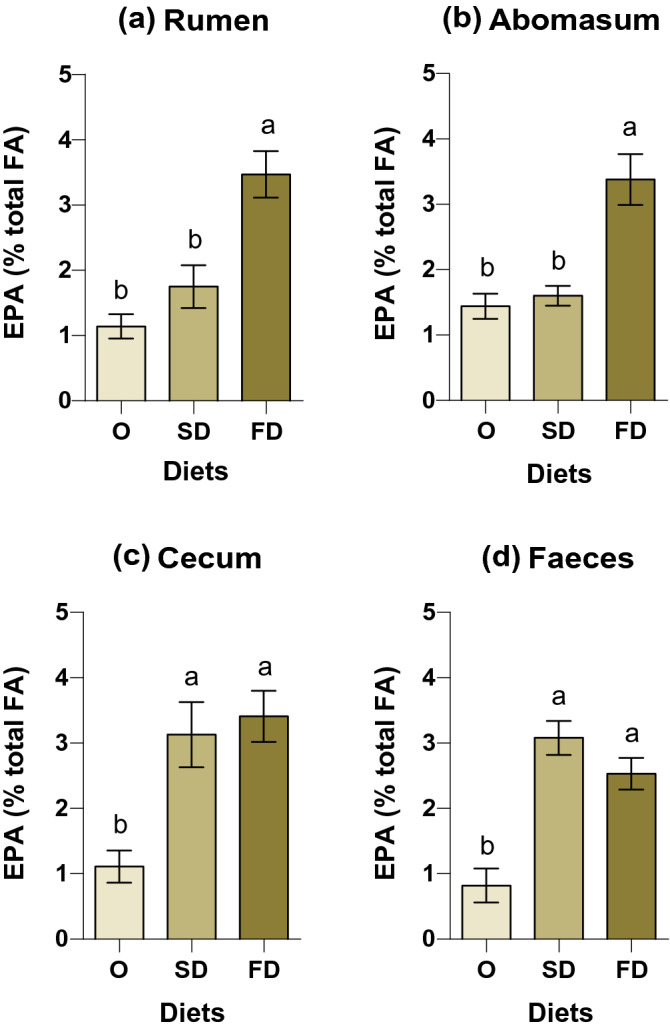
EPA content (% total fatty acids) in the (**a**) rumen content, (**b**) abomasal content, (**c**) cecal content, and (**d**) faeces. Diets are represented as follow: O (*Nannochloropsis sp*. oil), SD (spray-dried *N. oceanica*) and FD (freeze-dried *N. oceanica*). Least square means are presented in each bar with respective standard error of the mean. Different letters (a–c) in each bar indicate significant differences (*P* < 0.05) among means.

The proportion of EPA in the rumen was higher (*P* < 0.01) in the FD treatment (Fig. 1a), reaching 3.5% of total FA and dimethyl acetals (DMA)(1.6 mg/g DM), compared to SD and O treatments, which reached 1.8% and 1.1% of total FA + DMA, respectively. In the abomasal digesta the highest proportion (*P* = 0.001) of EPA (Fig. 1b) was found in FD treatment (3.4% of total FA + DMA) followed by O and SD treatments that averaged 2% of total FA + DMA. The EPA in cecum digesta and faeces (Fig. 1c,d, respectively) also differed (*P* < 0.001) among treatments, however both FD and SD treatments equally registered the higher content compared to O treatment.

**Figure 2:**
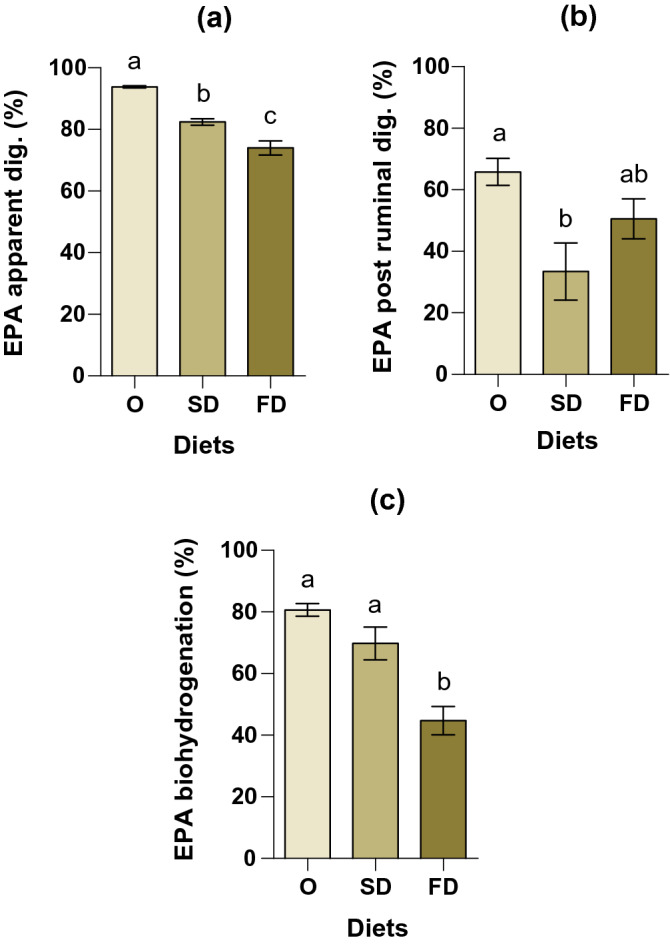
(**a**) EPA whole tract apparent digestibility (%), (**b**) EPA post ruminal digestibility (%) and (**c**) EPA biohydrogenation (%). Diets are represented as follow: O (*Nannochloropsis sp*. oil), SD (spray-dried *N. oceanica*) and FD (freeze-dried *N. oceanica*). Least square means are presented in each bar with respective standard error of the mean. Different letters in each bar (a–c) indicate significant differences (*P* < 0.05) among means.

The estimated whole tract EPA apparent digestibility was significantly different between diets (*P* = 0.002), being lowest in the FD diet (74.0% ± 2.31) fed lambs (Fig. 2a). The O treatment registered the highest EPA whole tract apparent digestibility (93.8% ± 0.38) and SD treatment presented an intermediate level (82.4% ± 1.02). The post-ruminal apparent digestibility also differed among diets (*P* = 0.012) being highest in O diet (65.8% ± 4.34) and lowest in SD diet (33.4% ± 9.31) (Fig. 2b), while FD did not differ from both O and SD diets. The estimated biohydrogenation of EPA (Fig. 2c) differed among the *Nannochloropsis* treatments (*P* < 0.001), increasing from 44.7% with FD diets to 69.8% with SD and 80.7% with O diet, respectively.

### Scanning electron microscopy (SEM) of *N. oceanica* biomass

**Figure 3:**
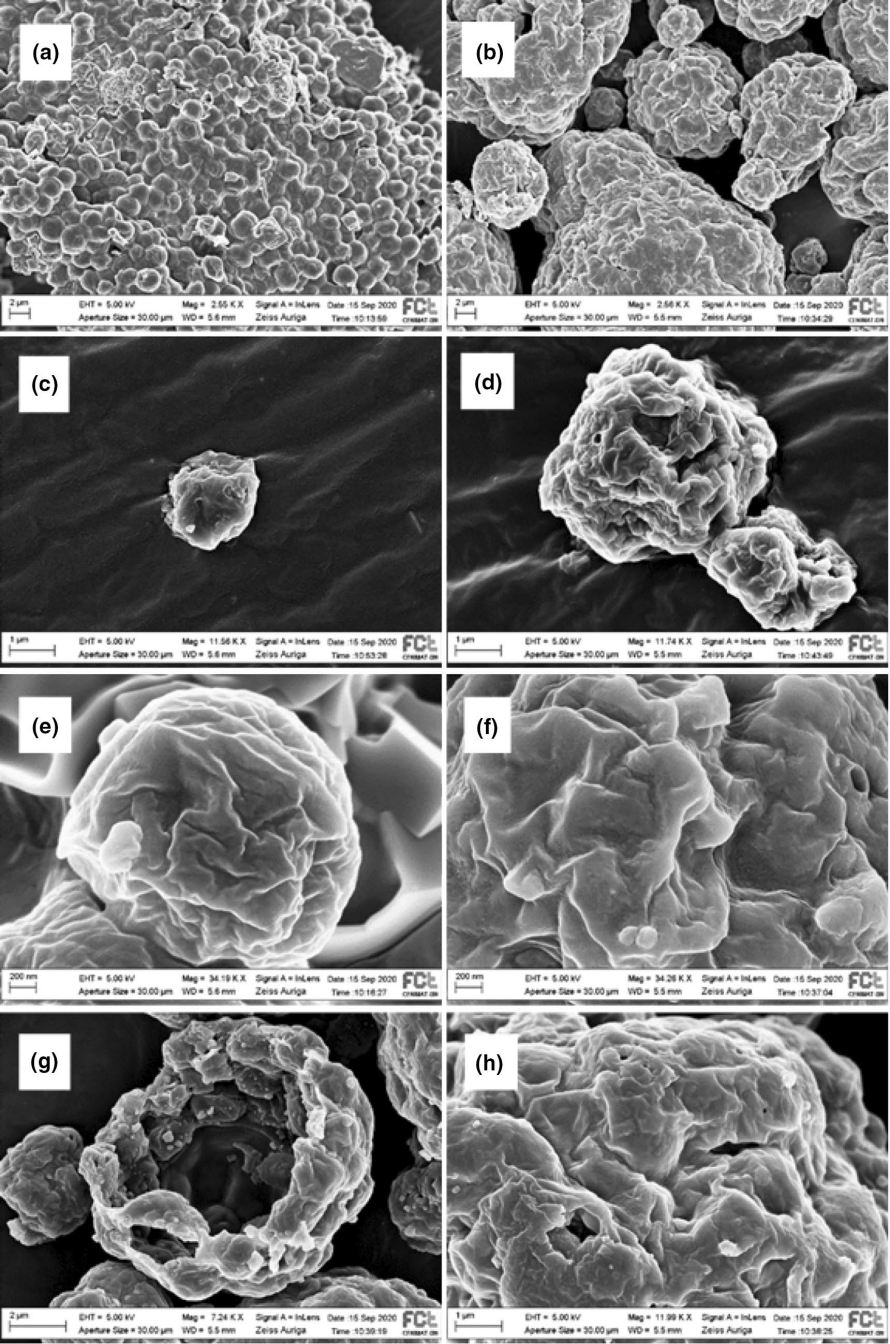
Scanning Electron Microscopy Focused Ion Beam (SEM–FIB) of freeze-dried (FD) and spray-dried (SD) *N. oceanica*. Stabilization in carbon matrix and gold–palladium coating. (**a**) Granules correspond to individualized FD *N. oceanica* cells; (**b**) Granules correspond to clusters of more than one SD *N. oceanica* cell; (**c**) Individual FD *N. oceanica* cell; (**d**) SD *N. oceanica* cells cluster; (**e**) FD *N. oceanica* cell wall appearance detail; (**f**) SD *N. oceanica* cell walls appearance in detail; (**g**) SD *N. oceanica* fragmented granule; (**h**) SD *N. oceanica* granule surface in detail.

The microscopic evaluation of the surfaces of SD and FD biomass incorporated in the diets revealed two different patterns (Fig. 3a–h). The FD biomass (Fig. 3a) consisted of a cluster of perfectly individualized cells (although organized in plate-like structures) that maintained an overall spherical shape. In contrast, SD biomass (Fig. 3b) consisted of a set of large amorphous granules consisting of clustered microalgae cells that loss the spherical structure, and thus they could not be easily individualized. Apparently, cell wall integrity was maintained in FD biomass but not in the SD form. Both FD and SD presented a wrinkled and slightly collapsed cell wall, although more severe alterations were found in the SD that presented fractured granules (Fig. 3g), and irregular shaped holes and cracks (Fig. 3h).

### Fatty acid composition of digesta contents

The total FA content, including DMA, and the partial FA sums in the rumen, abomasal and cecum digesta of lambs fed control (C) and *Nannochloropsis* supplemented diets are presented in Table 1. The complete FA and DMA profile are presented in the Supplementary Tables S1, S2 and S3.

**Table 1:** Total FA and dimethyl acetals content (mg/g DM) and FA partial sums (% total FA + DMA) in the rumen, abomasum, and cecum.

Item	Diets^1^				*P*-value
	**C**	**O**	**SD**	**FD**	
**Rumen**					
Total FA + DMA^2^	36.36^b^ ± 3.028	42.76^ab^ ± 4.461	38.80^b^ ± 1.545	45.79^a^ ± 1.143	0.004
SFA	49.28^ab^ ± 5.569	38.62^b^ ± 2.274	52.47^a^ ± 3.900	44.01^a^ ± 1.243	0.027
MUFA^3^	28.60 ± 4.662	30.40 ± 3.042	25.76 ± 2.648	30.35 ± 0.586	0.415
*trans*-MUFA	26.24 ± 4.830	26.20 ± 2.948	20.06 ± 2.434	21.90 ± 0.454	0.347
PUFA	12.18^b^ ± 1.279	17.75^a^ ± 1.569	12.66^b^ ± 1.657	17.70^a^ ± 1.387	0.012
DMA	4.87^a^ ± 0.328	3.96^b^ ± 0.171	3.82^b^ ± 0.284	3.44^b^ ± 0.307	0.030
C16:1 FA	0.23^c^ ± 0.030	1.78^b^ ± 0.177	2.75^b^ ± 0.648	5.93^a^ ± 0.436	< 0.001
BCFA	4.07^a^ ± 0.261	3.89^a^ ± 0.232	3.25^b^ ± 0.199	2.92^b^ ± 0.249	0.009
C18 FA^4^	66.62^a^ ± 0.601	52.15^b^ ± 3.302	47.82^b^ ± 0.397	43.81^c^ ± 0.1178	< 0.001
C18 BI^5^	29.52 ± 4.817	31.41 ± 3.182	24.18 ± 2.541	26.20 ± 0.556	0.316
Total C20 FA	1.35^c^ ± 0.127	7.04^b^ ± 0.791	7.67^b^ ± 0.223	9.08^a^ ± 0.269	< 0.001
C20:1 FA	0.06^c^ ± 0.034	0.58^b^ ± 0.088	1.06^a^ ± 0.162	0.64^ab^ ± 0.169	< 0.001
C20:2 FA	0.40^c^ ± 0.075	1.42^a^ ± 0.188	1.13^ab^ ± 0.132	0.77^b^ ± 0.126	< 0.001
C20:3 FA	0.12^c^ ± 0.021	1.59^a^ ± 0.259	0.72^b^ ± 0.171	0.99^ab^ ± 0.211	< 0.001
C20:4 FA	0.07^c^ ± 0.041	1.28^ab^ ± 0.372	1.15^b^ ± 0.212	2.07^a^ ± 0.190	< 0.001
**Abomasum**					
Total FA + DMA^2^	42.43^ab^ ± 6.529	33.74^b^ ± 4.053	49.24^a^ ± 3.165	47.32^a^ ± 5.112	0.043
SFA	50.50 ± 6.970	41.20 ± 2.226	50.74 ± 3.463	44.14 ± 0.899	0.133
MUFA^3^	30.35 ± 5.149	33.81 ± 2.966	30.05 ± 2.819	33.34 ± 0.643	0.655
*trans*-MUFA	19.26 ± 4.351	23.01 ± 3.199	17.12 ± 2.526	16.81 ± 0.644	0.303
PUFA	11.44^b^ ± 1.672	16.75^a^ ± 1.592	11.82^b^ ± 0.586	16.08^a^ ± 1.090	0.003
DMA	2.68^a^ ± 0.191	2.32^ab^ ± 0.228	1.91^b^ ± 0.154	2.08^ab^ ± 0.285	0.036
BCFA	3.87^a^ ± 0.283	3.77^ab^ ± 0.404	3.15^bc^ ± 0.195	2.74^c^ ± 0.248	0.028
C16:1 FA	0.48^c^ ± 0.047	2.75^b^ ± 0.278	3.75^b^ ± 0.500	6.96^a^ ± 0.438	< 0.001
C18 FA^4^	68.22^a^ ± 0.387	54.63^b^ ± 2.041	48.59^c^ ± 0.547	43.58^d^ ± 0.500	< 0.001
C18 BI^5^	21.08 ± 4.310	25.60 ± 3.320	20.51 ± 2.528	19.24 ± 0.946	0.336
Total C20 FA	2.35^c^ ± 0.418	7.83^b^ ± 0.596	9.16^ab^ ± 0.376	9.80^a^ ± 0.343	< 0.001
C20:1 FA	1.27 ± 0.428	0.76 ± 0.212	1.52 ± 0.230	1.03 ± 0.229	0.136
C20:2 FA	0.33^c^ ± 0.078	2.04^ab^ ± 0.255	2.08^a^ ± 0.178	1.47^b^ ± 0.180	< 0.001
C20:3 FA	0.15^c^ ± 0.019	2.10^a^ ± 0.246	1.35^b^ ± 0.249	1.23^b^ ± 0.177	< 0.001
C20:4 FA	0.07^d^ ± 0.14	0.36^c^ ± 0.049	0.79^b^ ± 0.064	1.62^a^ ± 0.116	< 0.001
**Cecum**					
Total FA + DMA^2^	42.02 ± 8.230	34.88 ± 6.677	28.42 ± 5.836	27.50 ± 3.459	0.376
SFA	58.81^ab^ ± 5.640	48.39^b^ ± 2.335	56.41^a^ ± 2.962	55.62^a^ ± 1.083	0.055
MUFA^3^	22.38^ab^ ± 4.454	26.85^a^ ± 2.943	17.58^b^ ± 2.217	18.32^b^ ± 0.747	0.048
*trans*-MUFA	21.42^ab^ ± 4.207	23.26^a^ ± 2.816	13.13^bc^ ± 2.284	12.07^c^ ± 0.785	0.003
PUFA	6.14^b^ ± 0.874	12.71^a^ ± 1.079	12.23^a^ ± 1.121	14.18^a^ ± 0.830	< 0.001
DMA	4.26 ± 1.354	4.91 ± 1.413	5.25 ± 0.886	5.01 ± 0.608	0.943
BCFA	6.79 ± 1.013	6.34 ± 0.838	7.91 ± 1.369	6.31 ± 0.370	0.706
C16:1 FA	0.24^d^ ± 0.047	1.21^c^ ± 0.177	2.56^b^ ± 0.328	4.44^a^ ± 0.559	< 0.001
C18 FA^4^	57.37^a^ ± 2.984	47.98^a^ ± 3.806	34.06^b^ ± 4.010	30.86^b^ ± 1.361	< 0.001
C18 BI^5^	17.54^ab^ ± 3.860	21.70^a^ ± 2.650	11.33^b^ ± 2.303	11.59^b^ ± 0.817	0.008
Total C20 FA	1.65^c^ ± 0.363	5.69^b^ ± 0.461	8.56^a^ ± 0.520	8.79^a^ ± 0.354	< 0.001
C20:1 FA	0.05^b^ ± 0.027	0.45^a^ ± 0.127	0.25^a^ ± 0.055	0.21^ab^ ± 0.041	0.003
C20:2 FA	0.61 ± 0.579	1.13 ± 0.285	0.81 ± 0.249	0.60 ± 0.183	0.479
C20:3 FA	n.d	1.08 ± 0.230	0.61 ± 0.178	0.94 ± 0.101	0.212
C20:4 FA	n.d	0.33^a^ ± 0.068	0.12^b^ ± 0.016	0.23^ab^ ± 0.056	0.012

Values are means ± standard error of the mean. Means within a row with different letters are significantly different (P < 0.05). ^1^Diets (or treatments): C, control; O, Control plus 1.2% *Nannochloropsis* sp. oil; SD, control plus 12.3% spray-dried *Nannochloropsis oceanica*; FD, control plus 9.2% freeze-dried *N. oceanica*. ^2^The sum of total fatty acids includes also dimethyl acetals (DMA). ^3^MUFA, includes fatty acids with undefined geometry ^4^C18 FA, fatty acids with a chain length of 18 carbon-atoms; ^5^C18 BI, C18 biohydrogenation intermediates, sum of total 18:1, 18:2, 18:3 and 18:0-oxo except the *c*9-18:1, *c*11-18:1, 18:2n-6 and 18:3n-3.

#### Ruminal digesta

Average FA and DMA content was highest in the FD (45.8 mg/g DM, *P* = 0.001), but it did not differ from O treatment (42.8 mg/g DM). The lowest total FA content (36.4 mg/g DM) was observed in the C treatment, but it did not differ from the O and SD treatments (Table 1). The proportion of saturated fatty acids (SFA) was affected by treatments (*P* = 0.027), being highest in the SD and lowest in the O treatment (52.5% and 38.6% of total FA + DMA, respectively). Neither monounsaturated FA (MUFA) nor *trans*-MUFA proportions in the rumen differed among treatments (*P* = 0.415 and *P* = 0.347, respectively), as there were no differences in total C18 biohydrogenation intermediates (BI). On the contrary, PUFA were higher in the O and FD treatments (averaging 17.7% of total FA + DMA) comparing to C and SD treatments (averaging 12.6% of total FA + DMA). The branched-chain fatty acids (BCFA) were higher in the rumen of lambs fed C and O and lower in those fed SD and FD diets. The highest proportion of the total C20 FA was found in the FD treatment (9.1% of total FA + DMA), followed by SD and O treatments (7.7% and 7.1% of total FA + DMA, respectively), and the C showed the lowest proportion (1.4% of total FA + DMA) (Table 1).

#### Abomasal digesta

Average FA and DMA content was lowest for animals fed O diet (33.7 mg/g DM), although not different from animals fed C diet (42.4 mg/g DM), while the highest content was observed in animal fed SD (49.2 mg/g DM), but it did not differ from those fed FD and C diets. Contrary to what was observed for the rumen contents, SFA did not differ among treatments (*P* = 0.133), but similarly, to what was observed in the rumen, the proportion of MUFA, *trans*-MUFA and C18 BI did not differ (*P* > 0.05) among treatments. The proportion of PUFA was higher (*P* = 0.003) in animals fed O and FD diets (averaging 16.4% of total FA + DMA) compared to C and SD diets, (averaging 11.6% of total FA + DMA). Similarly, to what was observed in the rumen, the total C18 FA differed among treatments (*P* < 0.001), being highest and lowest in the C (68.2%) and FD fed lambs (43.6%), respectively. The total DMA proportion was highest in C (2.7%) and lowest in SD treatments (1.9%) while O and FD presented intermediate proportions. The highest BCFA proportion was also observed in C diet (4.0% of total FA + DMA) but the lowest proportion was observed in FD diet (2.7% of total FA + DMA). The highest proportion of total C20 was observed in FD diet (9.8% of total FA), while in C diet it only reached 2.4% of total FA. There were also differences (*P* < 0.001) among treatments for the sums of C20:2, C20:3 and C20:4.

#### Caecal digesta

Average FA + DMA content did not differ (*P* = 0.376) among treatments (Table 1). Following the same trend that was verified in the ruminal content, the proportion of SFA showed a tendency (*P* = 0.055) for being higher in the cecum of C, SD, and FD lambs (averaging 57.0% of total FA + DMA) than in the O fed lambs (48.4% total FA + DMA). Contrary to what was observed in both rumen and abomasum, the proportion of MUFA and *trans*-MUFA in cecum differed among treatments (*P* < 0.05), being the lowest proportions found in FD and SD fed animals. Moreover, total C18 FA and C18 BI differed among treatments (*P* < 0.001 and *P* = 0.008, respectively) being highest in the C and O treatment and lowest in the SD and FD treatments. In the cecum, neither total DMA nor BCFA differed among treatments (*P* > 0.05). PUFA presented higher proportions (*P* < 0.001) in *Nannochloropsis* fed lambs (averaging about 13% of total FA) compared to C fed animals that only showed 6.1% of total FA. The proportions of total C20 FA were higher (*P* < 0.001) for FD and SD treatments (8.8 and 8.6% of total FA + DMA, respectively) compared to O treatment (5.7% of total FA + DMA).

### Biohydrogenation of C18 PUFA in the rumen

**Figure 4:**
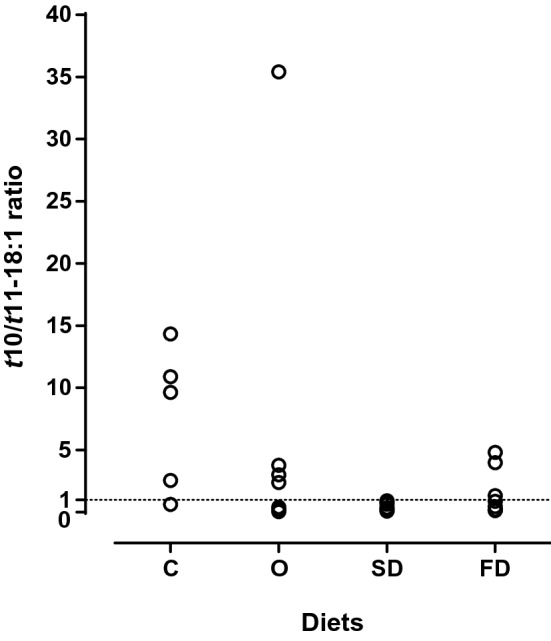
Dispersion of individual values of *t*10/*t*11-18:1 ratio in the rumen of lambs fed experimental diets. Diets are represented as follow: C (Control), O (*Nannochloropsis sp*. oil), SD (spray-dried *N. oceanica*) and FD (freeze-dried *N. oceanica*). Least square means are presented in each point.

Diets were not formulated considering the C18 FA, nevertheless the content of the C18 FA (g/kg DM) were similar among diets. Also, to better evaluate the effect of *Nannochloropsis* supplementation on the biohydrogenation of C18 PUFA in the rumen, the profile of C18 FA was expressed as a percentage of total C18 FA (Table 2). Although the total C18 FA (mg/g DM) only tended to differ (*P* = 0.080) across treatments, several C18 BI differed statistically among treatments. The major BI were the *t*10-18:1 and *t*11-18:1. The *t*11-18:1 was higher with *Nannochloropsis* supplemented diets (averaging 14.1% of total C18 FA) than with the C diet. On the contrary, the *t*10-18:1 tended (*P* = 0.057) to be highest in C and lowest in SD treatments. The ratio between *t*10-18:1 and *t*11-18:1 (*t*10/*t*11-18:1 ratio) was highest in the C treatment (7.6), although it did not differ from O treatment (6.5) due to one sample with a very high *t*10/*t*11-18:1 ratio (Fig. 4), and it was lowest in the SD and FD treatments (0.47 and 1.69, respectively).

**Table 2:** Total C18 fatty acid content (mg/g DM) and composition (% of total C18) in the rumen and biohydrogenation (BH) indicators.

Item	Diets^1^				*P*-value
	**C**	**O**	**SD**	**FD**	
Total C18 FA^2^	24.26 ± 2.185	22.15 ± 2.41	18.57 ± 0.81	20.06 ± 0.49	0.080
18:0	39.86^a^ ± 7.145	21.86^b^ ± 2.751	35.28^ab^ ± 6.744	20.76^b^ ± 1.889	0.034
*t*6/*t*7/*t*8-18:1	1.32 ± 0.312	2.24 ± 0.305	2.00 ± 0.350	2.36 ± 0.171	0.056
*t*9-18:1	0.61^b^ ± 0.221	1.43^a^ ± 0.203	1.60^a^ ± 0.252	1.69^a^ ± 0.107	0.002
*t*10-18:1	20.80 ± 6.187	18.64 ± 5.655	6.66 ± 2.089	12.4 ± 2.718	0.057
*t*11-18:1	3.46^b^ ± 1.078	11.94^a^ ± 3.550	15.48^a^ ± 2.911	14.92^a^ ± 3.168	0.001
*t*12-18:1	1.12^c^ ± 0.113	1.29^bc^ ± 0.253	2.17^a^ ± 0.206	2.10^ab^ ± 0.315	0.001
*t*13/*t*14/*c*9-18:1	10.83 ± 1.259	12.54 ± 1.041	12.05 ± 1.880	14.66 ± 1.018	0.143
*t*15-18:1	0.84^ab^ ± 0.101	0.46^b^ ± 0.167	1.22^a^ ± 0.177	0.98^ab^ ± 0.183	0.036
*c*11-18:1	1.31^c^ ± 0.248	2.05^ab^ ± 0.239	1.97^b^ ± 0.169	2.64^a^ ± 0.247	0.010
*c*12-18:1	0.52^b^ ± 0.097	0.51^b^ ± 0.110	1.10^a^ ± 0.199	0.82^a^ ± 0.064	0.012
*c*13-18:1	0.08^b^ ± 0.015	0.09^b^ ± 0.015	0.14^a^ ± 0.016	0.13^ab^ ± 0.024	0.026
*t*16/*c*14-18:1	0.52 ± 0.099	0.29 ± 0.145	0.80 ± 0.161	0.53 ± 0.148	0.169
*c*15-18:1	0.41^ab^ ± 0.113	0.58^a^ ± 0.127	0.24^b^ ± 0.041	0.56^ab^ ± 0.168	0.038
*c*16-18:1	0.13^b^ ± 0.013	0.20^a^ ± 0.020	0.19^ab^ ± 0.034	0.25^a^ ± 0.037	0.003
*t,t*-18:2	0.13 ± 0.033	0.36 ± 0.138	0.10 ± 0.018	0.13 ± 0.027	0.224
*t*9,*c*12-18:2	0.11^b^ ± 0.018	0.29^ab^ ± 0.092	0.29^ab^ ± 0.105	0.47^a^ ± 0.112	0.006
*t*11,*c*15/*t*10,*c*15-18:2	0.66^b^ ± 0.155	2.97^a^ ± 0.742	0.55^b^ ± 0.147	1.48^a^ ± 0.339	0.007
18:2n-6	13.52 ± 1.681	15.56 ± 1.256	10.43 ± 2.532	15.84 ± 1.957	0.279
*c*9,*t*11-CLA	n.d	0.18 ± 0.022	0.40 ± 0.108	0.27 ± 0.061	0.102
*t*,*t*-CLA	0.26 ± 0.015	0.23 ± 0.036	0.37 ± 0.072	0.73 ± 0.267	0.119
18:3n-3	2.53^b^ ± 0.215	3.52^a^ ± 0.269	3.73^a^ ± 0.114	3.58^a^ ± 0.169	0.001
oxo-18:0	0.97^b^ ± 0.362	2.95^a^ ± 0.660	3.23^a^ ± 0.554	2.78^a^ ± 0.670	0.006
*t*10/*t*11-18:1 ratio	7.61^a^ ± 2.594	6.46^ab^ ± 4.859	0.47^b^ ± 0.124	1.69^b^ ± 0.725	0.022
**BH indicators**					
BH-18:2n-6	75.79 ± 3.101	74.14 ± 2.013	82.14 ± 4.334	71.03 ± 3.580	0.270
BH-18:3n-3	79.11^a^ ± 1.768	72.97^b^ ± 1.598	64.04^c^ ± 1.002	70.27^b^ ± 1.403	< 0.001
Completeness^3^	55.03^a^ ± 8.813	28.68^c^ ± 2.438	46.97^ab^ ± 7.78	31.91^bc^ ± 2.11	0.018

Values are means ± standard error of the mean. Means within a row with different letters are significantly different (P < 0.05). ^1^Diets (or treatments): C, control; O, Control plus 1.2% *Nannochloropsis* sp. oil; SD, control plus 12.3% spray-dried *Nannochloropsis oceanica*; FD, control plus 9.2% freeze-dried *N. oceanica*. ^2^C18 FA, Fatty acids with 18 carbon chain length. ^3^C18 biohydrogenation completeness (%) that was estimated considering the maximum 18:0 in the rumen and assuming a complete biohydrogenation of the C18 FA from the diet. n.d – not detected.

Other rumen C18 BI differed (*P* < 0.05) among treatments, namely *t*9-18:1, *c*16-18:1, and *t*9,*c*12-18:2 were highest in FD, and the *t*12-18:1, *c*12-18:1, *c*13-18:1 and oxo-18:0 were highest in SD, whereas the C treatment presented the lowest proportions of these BI (Table 2). No differences among treatments were observed for 18:2n-6, but the 18:3n-3 was 43% higher with *Nannochloropsis* supplemented diets than with C diet (*P* = 0.001). In contrast, the 18:0 was about two folds higher with C diet (40% of total C18 FA) than with FD diet (21%), and the O and SD diets presented intermediate values, i.e., 22% and 35% of total C18 FA, respectively (Table 2).

Consistent with these results, the estimated biohydrogenation of 18:2n-6 did not differ (*P* = 0.133) among treatments (Table 2), whereas the biohydrogenation of 18:3n-3 was highest in C and lowest in SD group (*P* < 0.001). Also, the estimated biohydrogenation completeness differed (*P* = 0.01) among treatments, reaching the highest value with C (55.0%) and the lowest with O and FD treatments (averaging 30.3%), while the biohydrogenation completeness in SD did not differ from the other treatments.

### Fermentation parameters, protozoa counting, and rumen mucosa evaluation

Rumen pH averaged approximately 6.0 ± 0.26 in animals consuming different diets and did not differ (*P* = 0.782) among them (Table 3). Total volatile fatty acids (VFA) concentration, which averaged 222 mmol/L, the molar proportions of linear chain VFA (2:0, 3:0, 4:0, 5:0 and 6:0) and the branched-chain VFA (*iso*-4:0 and *iso*-5:0) did not differ among treatments (*P* > 0.05).

**Table 3:** Rumen pH, volatile fatty acid (VFA) concentration and molar proportion, and mucosa variables of lambs fed experimental diets.

Item	Diets^1^				*P*-value
	**C**	**O**	**SD**	**FD**	
Rumen pH	5.7 ± 0.27	6.0 ± 0.23	6.0 ± 0.26	5.8 ± 0.26	0.782
Total VFA (mmol/L)	199 ± 31.6	246 ± 45.9	233 ± 63.8	211 ± 31.2	0.847
VFA (mol/100 mol)					
2:0	43.4 ± 3.32	47.9 ± 4.06	50.2 ± 2.10	50.5 ± 2.54	0.329
3:0	26.3 ± 2.82	26.9 ± 2.0	23.6 ± 0.75	24.9 ± 1.97	0.370
i-4:0	6.8 ± 2.09	6.0 ± 3.01	6.7 ± 1.55	5.6 ± 1.25	0.928
4:0	14.3 ± 1.49	15.6 ± 2.24	13.3 ± 1.50	12.7 ± 0.79	0.564
i-5:0	3.1 ± 1.17	2.0 ± 0.75	3.3 ± 0.56	2.4 ± 0.58	0.518
5:0	4.7 ± 1.24	2.9 ± 0.60	2.5 ± 0.54	3.3 ± 0.58	0.417
6:0	1.4 ± 0.53	0.9 ± 0.29	0.5 ± 0.09	0.7 ± 0.27	0.206
Rumen mucosa variables					
Papillae length (mm)	5.30 ± 0.37	5.03 ± 0.33	5.76 ± 0.78	4.33 ± 0.30	0.125
Epithelium thickness (µm)	195 ± 20.3	173 ± 20.2	182 ± 16.8	167 ± 7.5	0.558
Stratum corneum thickness (µm)	53.6 ± 6.91	47.1 ± 3.87	52.7 ± 3.85	45.9 ± 2.39	0.410
Greyscale value	59.3 ± 4.86	61.8 ± 3.56	61.7 ± 6.15	56.1 ± 620	0.867

Values are means ± standard error of the mean. ^1^Diets: C, control; O, Control plus 1.2% *Nannochloropsis* sp. oil; SD, control plus 12.3% spray-dried *Nannochloropsis oceanica*; FD, control plus 9.2% freeze-dried *N. oceanica.*

Most of the lambs were defaunated and only 3 presented countable rumen ciliate protozoa. Two lambs consuming SD diet had counts of 2.3 × 10^6^ and 7.5 × 10^5^ ciliate cells per mL and one animal consuming O diet had a count of 2.3 × 10^6^ ciliate cells per mL. *Isotricha* genus was only found in one animal fed SD diet (1.0 × 10^5^ cells per mL). *Epidinium* were only found in the lamb fed O diet (1.0 × 10^6^ cells per mL). *Entodinium* sp were found in three lambs, two from the SD group having 2.2 × 10^6^ and 7.5 × 10^5^ cells per mL and one from the O fed group having 1.3 × 10^6^ cells per mL.

Regarding the evaluation of rumen mucosa integrity in what to concerns to the presence of parakeratotic lesions, none of the histometric indicators (i.e., papillae length, epithelium thickness, and *stratum corneum* thickness) differed among treatments (*P* > 0.120) as shown in Table 3. Image greyscale values from ruminal mucosa digital photograph processing did not differ among treatments (*P* = 0.867) as well.

## Discussion

In a previous in vitro study^7^, we found that EPA disappearance from *N. oceanica* dried biomass, after incubation with rumen fluid for 24 h, was reduced compared with non-esterified EPA (unprotected EPA) and that the EPA disappearance was 25% lower when *N. oceanica* biomass was FD than SD. We hypothesised that protection against biohydrogenation was somehow related to *N. oceanica* cell wall’s structure and that the freeze-drying would better preserve the integrity of cell walls^7^. The present experiment was designed to confirm in vivo our previous findings related to the higher capacity of FD *N. oceanica* to decrease EPA disappearance on in vitro rumen batch incubations compared to SD *N. oceanica*. Thus, we designed the lamb diets to provide similar amounts of EPA, via free *Nannochloropsis* sp. oil or *N. oceanica* biomass, either SD or FD. However, after diet sampling, the final EPA content was slightly lower in FD diet compared to SD or O diets. This could be related with losses during handling, feed preparation or differences among slurry batches. Despite that, the results confirmed that the EPA from FD biomass was better protected from rumen biohydrogenation than SD biomass. The estimates of biohydrogenation extent (disappearance) of EPA in the rumen also confirmed that FD *N. oceanica* was more protected from biohydrogenation, as EPA biohydrogenation of FD was only 45% compared to fairly high values found with SD (70%) and O (81%). Consistently the concentration of the EPA in the rumen of lambs fed FD was about 50% higher than lambs fed SD or O diets.

These results reinforce the hypothesis that the *N. oceanica* cell wall structure was differently affected by the slurry drying method allowing the lipids to be distinctly exposed to rumen microbes. Indeed, SEM images confirmed that *N. oceanica* cells in FD biomass were better preserved than SD biomass. Differential effects of spray-drying or freeze-drying on *Chlorella* and *Spirulina* microalgae biomass microstructure evaluated by SEM were previously reported^8^. This author also observed that SD particles consisted of multicellular spheres with a void space in the middle. However, the Chlorella and Spirulina FD particles consisted in sheets of cells that were no longer spherical but adhered together in a linear fashion, which differed from what we presently found. The freeze-dried method can cause damage to cells, as intracellular water expands upon freezing^10^, but this might be highly variable with the freezing conditions and with microalgae species. Nevertheless, the impact of the freeze-dried process on the cell wall structure seems to be less drastic compared with spray-dried as previously discussed^7^. The changes on the surface of *N. oceanica* biomass morphology reported here evidenced that FD maintains a better overall cell structure (shape and integrity), while SD suffered more severe shape alterations, including the presence of cracks and holes. This integrity loss of SD *N. oceanica* biomass might have compromised the ability to keep the EPA inside the cell structures, exposing it to microbial metabolization. This could explain why rumen biohydrogenation of EPA with the SD treatment was much higher than FD’s and similar to that found when free *Nannochloropsis* oil was used.

Ruminal biohydrogenation of EPA and DHA from several experiments with fish oil or marine microalgae averaged 80% as reviewed by Doreau and co-workers^3^. This value is similar to that found for the biohydrogenation of EPA from *Nannochloropsis* oil, which supports the evidence of the effective protection against microbial attack in the rumen offered by FD *N. oceanica* biomass. Considering that the *Nannochloropsis sp.* contains relevant amounts of *c*9-16:1 and 20:4n-6, both were also partially protected from ruminal metabolization with the FD *N. oceanica*. So, the ruminal biohydrogenation of 20:4n-6 was lowest in FD group and highest in lambs fed *Nannochloropsis* free oil. The highest proportion of 20:2 and 20:3 intermediates, likely to be formed from biohydrogenation of EPA (20:5n-3) and 20:4n-6, was found in the O group. These results suggest that in oil, FA are more exposed to rumen microbes than in SD and FD *N. oceanica* biomass. Moreover, the formation of a large range of C20 intermediates from the ruminal biohydrogenation of unesterified EPA using deuterated (d5-20:5n-3)^7^ and non-deuterated forms^11^ was already demonstrated in vitro.

An efficient lipid-protection supplement needs to allow lipid release during abomasal passage and further digestion and absorption in the intestine^6^. We did not evaluate microalgae cell disruption in the abomasum, but the highest proportion of EPA in the abomasal digesta was found in FD *N. oceanica* fed animals, similar to what was observed in the ruminal digesta. Also, the proportions of total SFA, MUFA and PUFA in abomasal digesta were in the same range to those found in the rumen content. Indeed, the FA composition of abomasal contents have been shown to remain similar to that leaving the rumen^12^ because medium‐ and long‐chain FA are minimally absorbed or modified in the omasum or abomasum^13^. Additionally, the acid environment at abomasum should help disruption of *N. oceanica* cell walls and promote intracellular lipids to get released. In fact, low pH solution was reported to increased porosity of cell wall and help lipid extraction from several microalgae, including *Nannochloropsis sp*.^14^.This microalgae cell disintegration will allow the digestion and absorption of EPA and other nutrients in the intestine. However, the high levels of EPA in both cecum and faeces of animals fed *N. oceanica* biomass, independently of the drying method, suggests that EPA was not completely released and absorbed in the small intestine.

Compared to the literature our estimates of post-ruminal apparent digestibility of EPA are quite low for SD (33%) and FD (51%) treatments but similar to previous reported values for O treatment (66%). In sheep the apparent intestinal digestibility of EPA supplied by fish oil ranged between 73 and 89%^15^. Moreover, Doreau et al. revising the literature found an average apparent intestinal digestibility of EPA of 80%^3^. Our figures are only crude estimates but clearly point to large difference of EPA *post*-ruminal apparent digestibility of *N. oceanica* biomass than free oil, which is consistent with the protection against EPA metabolization in the rumen. However, contrary to what could be expected the *post*-ruminal apparent digestibility was lower with SD than with FD biomass. Thus, despite the EPA in the SD particles being more exposed to rumen metabolism than in FD particles, after rumen passage, the EPA remaining in the SD particles seems less available to intestinal digestion than that remaining on FD particles.

Dietary supplementation of EPA or DHA-enriched products, especially from marine origin, affects the ruminal biohydrogenation of both 18:2n-6 and 18:3n-3 by disturbing the rumen microbial population and inhibiting the final biohydrogenation reductive step, resulting in the accumulation of *trans*-18:1 isomers and reduction of 18:0 in the rumen^16–18^. Indeed, several studies reported that dietary supplementation of fish oil or microalgae lipid extracts decreased the proportion of 18:0 and increased the *trans*-18:1 in the rumen and in the duodenal flows^18–21^. Consistently with the literature, the proportion of 18:0 in the rumen was highest in the C treatment and decreased in *Nannochloropsis* fed lambs (Supplementary Table S1), even when expressed in percentage of total C18 FA. However, in SD fed lambs, neither the 18:0 nor C18 biohydrogenation completeness in the rumen differed from the C group. In vitro incubations with unesterified DHA or DHA-microalgae suggested that the DHA is the active component that promotes incomplete biohydrogenation of C18 PUFA^22^ and this might also be extrapolated to EPA^11^. Thus, inhibition of the completeness C18 biohydrogenation is expected to be directly related to EPA release in the rumen, being lowest in the more protected FD biomass, intermediate in SD biomass and highest in the free oil. However, our results do not fit this pattern, as this inhibition of the last reductive step of the biohydrogenation was highest for both O and FD and lowest for SD, suggesting that other factors besides the amount of EPA released in the rumen might modulate this effect. Factors like the type of metabolites formed, and the total PUFA concentration might have a role in disturbing the bacteria responsible for the last hydrogenation step to 18:0^19^.

Several individual C18 BI differed among treatments, with a particular interest in *t*11-18:1 and *t*10-18:1. The *t*11-18:1 is often the main *trans*-18:1 to accumulate in the rumen of animals fed forage-based diets but occasionally, a shift toward the formation of *t*10-18:1 at the expense of *t*11-18:1 (*t*10-shift, with *t*10/*t*11-18:1 ratio > 1) is observed, particularly in high-concentrate diets supplemented with vegetable oils and/or low rumen pH^23^. In the present experiment, the *t*10-shift was evident (i.e., *t*10/*t*11-18:1 ratio ≈ 7.61) in C treatment. As there were no differences in rumen pH (≈ 5.9) among treatments and the basal diet was the same of the C, it was expected that *t*10-shift observed in the C was also present in the *Nannochloropsis* fed animals. But surprisingly, lower *t*10/*t*11-18:1 ratios were observed in animals fed the *Nannochloropsis* dried biomasses compared to C diet, while *Nannochloropsis* sp. extracted oil did not differ among groups but showing a large individual variability. Large individual susceptibility of lambs and bulls to the *t*10-shift has been often observed, although not well understood^23, 24^. Lipid supplementation often favours the *t*10-shift, particularly when supplementing low-forage diets^25, 26^. Marine lipids supplementations have been identified as one of the triggers of milk fat depression in dairy cows, which in great part could be explained by the rumen *t*10-shift occurrence^27^. Nevertheless, the effect of marine lipid is often determined by the composition of typical dairy or finishing diets. Data on supplementation of high-forage, with marine lipids are more scarce and do support that marine lipids are not relevant as inducer of the *t*10-shift^20, 28^. The notable decrease in the *t*10/*t*11-18:1 ratio with the inclusion of *N. oceanica* biomass was due to a large increase of *t*11-18:1 accumulation and tend to decrease the accumulation of *t*10-18:1, indicating that *N. oceanica* biomass was effective in deviating the C18 biohydrogenation pathways toward the *t*11-18:1 production. A potential explanation for such positive effect of *N. oceanica* biomass in mitigating the *t*10-shift could be linked to the additional vitamin E added in the *Nannochloropsis* diets. Indeed, vitamin E has been suggested to inhibit the *t*10-shift^29^, but such preventive effect was not confirmed in beef cattle or lambs, as evaluated by *trans*-18:1 deposition in the tissues^30, 31^. Moreover, the O diet also included the same additional vitamin E content than SD and FD diets and did not offer such a clear mitigation of the *t*10-shift, suggesting a direct effect of *N. oceanica* biomass. The occurrence of the *t*10-shift in finishing ruminants is a major constraint of the strategies based on lipid supplementation designed to improve the nutritional value of meat^23^, and this promising effect of *N. oceanica* biomass might constitute a clue towards a novel approach to reduce *t*10-shift occurrence in animals fed high-concentrated diets.

Other major C18 BI were affected by treatments as the coeluted peak of *t*11,*c*15-/*t*10,*c*15-18:2 that was higher in lambs fed O and FD diets than in those fed C and SD diets. Both *t*11,*c*15-18:2 and *t*10,*c*15-18:2 are intermediates from the biohydrogenation of 18:3n-3^32^, thus the low levels in the rumen of animals fed SD might be explained by the lower biohydrogenation estimated for 18:3n-3 compared with the other diets. The low levels in C group might be explained by the high C18 completeness, as previously discussed. Indeed, supplementation with microalgae has been reported to favour the accumulation of *t*11,*c*15-18:2 in rumen fluid of cows^17^ and ewes^21^. Other studies also reported that fish oil increases the ruminal outflow of *t*11,*c*15-18:2^20, 33^ due to incomplete biohydrogenation of 18:3n-3.

No differences among treatments were found on 18:2n-6 or in its estimated biohydrogenation. Thus, contrasting to what was observed for 18:3n-3, diets did not affect the initial steps of 18:2n-6 biohydrogenation. Similar results have been observed on the in vitro incubations with rumen fluid and SD or FD *N. oceanica*^7^. Other authors also did not find effects of dietary microalgae inclusion on the concentration of 18:2n-6 in the rumen^17, 34, 35^.

Globally, our results are consistent with the evidence that supplementation with marine lipid sources rich in long-chain PUFA affects the biohydrogenation of C18 PUFA. However, the differences in C18 biohydrogenation intermediates among treatments indicate changes in the predominance of competing biohydrogenation pathways and eventually of the rumen microbiota. Although complete rumen microbiome analysis is not available, protozoal counts showed that many lambs were defaunated. However, because defaunation was observed in animals from both Control and *Nannochloropsis* groups, any potential toxic effect of the microalgae was excluded, also the basal diet was a common diet as presented in Table 4. Rumen bacteria are known to play the main role in biohydrogenation; however, the relationship between rumen ciliate protozoa and biohydrogenation of C18 FA has been reported^36, 37^.

**Table 4:** Ingredients, chemical composition, and fatty acid profile of the experimental diets.

Item	Diets^1^			
	**C**	**O**	**SD**	**FD**
Ingredients (g/kg DM)				
Barley	390	385	342	354
Soybean meal	170	168	149	154
Dehydrated alfalfa	400	395	351	363
Freeze dried *N. oceanica*	–	–	–	92
Spray dried *N. oceanica*	–	–	123	–
Nannochloropsis sp. Oil	–	12	–	–
Calcium carbonate	13	13	11	12
Sodium bicarbonate	20	20	18	18
Marine salt	4	4	4	4
Premix^2^	3	3	3	3
Vitamin E	–	1.67	1.67	1.67
Chemical composition (g/kg DM)				
DM	904	907	902	907
Crude Protein	191	183	206	211
Ether Extract	13.3	32.5	35.2	33.9
NDF	267	267	232	290
ADF	173	174	150	167
ADL	30.3	28.1	26.8	31.2
Sugar	73.2	69.4	65.5	67.9
Starch	277	279	247	227
Crude Energy (kJ/100 g)	-	-	-	-
Ash	84.6	89.9	114.1	90.8
Total Fatty acids (g/kg DM)	13.7	20.6	19.5	20.2
FA profile (g/kg DM)				
14:0	n.d	0.26	0.47	0.46
16:0	3.49	4.74	4.66	5.27
16:1*c*9	0.07	1.06	1.99	1.66
17:0	0.09	0.07	n.d	n.d
18:0	0.56	0.61	0.49	0.67
18:1*c*9	2.55	2.99	2.30	2.77
18:1*c*11	0.10	0.19	0.14	0.14
18:2n-6	5.62	6.49	5.50	5.88
18:3n-3	1.22	1.40	0.98	1.30
20:4n-6	n.d	0.53	0.72	0.64
20:5n-3 (EPA)	n.d	2.14	2.20	1.39
22:0	n.d	0.12	0.07	n.d

^1^Diets (or treatments): C, control; O, Control plus 1.2% *Nannochloropsis* sp. oil; SD, control plus 12.3% spray-dried *Nannochloropsis oceanica*; FD, control plus 9.2% freeze-dried *N. oceanica*. ^2^Premix composition: Vitamin A, 4,000,000 UI; Vitamin D3, 1,100,000 UI; Vitamin E, 7500 mg/kg; Vitamin B1, 250 mg/kg; Vitamin B2 250 mg/kg; Zinc, 35,000 mg/kg; Iron, 12,500 mg/kg; Manganese, 17,500 mg/kg; Iodine, 200 mg/kg; Cobalt 250 mg/kg; Selenium, 100 mg/kg; Magnesium oxide (excipient) 40,000 mg/kg. n.d. – not detected.

Incorporation of supplements containing very long-chain PUFA, as those supplied by fish oil or microalgae, can affect rumen fermentative activity often increasing propionic acid molar proportion^38^. In our experiment, none of diets containing *Nannochloropsis* sources affected the rumen fermentation parameters, probably because the amount incorporated in the diet was not high (1.2% of oil or microalgae biomass supplying equivalent amount of EPA). Nevertheless, other authors reported increases on VFA and propionate in goats fed with dietary inclusions of *Nannochloropsis oculata* biomass as low as 0.5%^39^. The effects of *Nannochloropsis* microalgae on rumen fermentation might be dependent on the type of basal diet as reported for *Nannochloropsis salina*, that induced larger effects on rumen fermentation in continuous flow fermenters when fed with forage basal diet and minor effect when fed with a concentrate basal diet^40^. Thus, differences in experimental conditions, including animal species, basal diets, or even in intrinsic differences in the nutrient composition and cell wall structure of each *Nannochloropsis* species might result in different effects on rumen fermentation.

In south-western Europe, light lambs are usually finished for few weeks after weaning with high-energy diets and slaughter with up to 25 kg of liveweight. In these conditions a high incidence of rumen mucosa lesions including ruminal parakeratosis are observed and thus we routinely evaluate the rumen mucosa lesions in our lamb experiments^41–43^. Parakeratosis of rumen mucosa is characterized by an accumulation of layers of keratinized, nucleated squamous epithelial cells and excessive sloughing of the epithelium, increased thickness of the *stratum corneum* and consequent dark brown coloration of the mucosa^44–46^. In the present study, none of the histological parakeratosis indicators nor colour greyscale values differed among treatments, which indicates the absence of parakeratotic lesions. Despite neither the histometric parameters nor the pH differed among diets (*P* > 0.05), a negative Pearson correlation between *stratum corneum* thickness and ruminal pH was found (*r* =− 0.38, *P* = 0.043). The inclusion of 35–40% of dehydrated alfalfa in lamb diets could have contributed to the lack of differences, although rumen lesions in lambs have been reported using similar diets^42^. These results indicate that dietary inclusion of *Nannochloropsis* biomass in lamb finishing diets does not influence the occurrence of ruminal wall lesions.

We conclude that the drying method applied to *N. oceanica* strongly influences powder architecture and cell wall integrity and consequently the degree of EPA protection against rumen microbes. Indeed, we confirmed that freeze-drying has an advantage over spray-drying in preserving *N. oceanica* cell wall. Thus, FD *N. oceanica* can constitute a better source of ruminal protected-EPA comparing to SD *N. oceanica*, once higher EPA levels were found in the rumen and abomasum, indicating a better escape against ruminal biohydrogenation. However, EPA was also found in cecum content and faeces, suggesting that its absorption at the small intestine was not totally efficient.

Moreover, the supplementation of high-concentrated diets with *Nannochloropsis* microalgae affected the biohydrogenation of C18 fatty acids. The most notable effect was the deviation from the *t*10 biohydrogenation pathways to the *t*11 pathways, resulting in the higher abundance of *t*11-18:1 over *t*10-18:1 in the rumen of lambs fed *N. oceanica*. Also, at this level of *Nannochloropsis* incorporation, no disturbances were found in fermentable parameters nor ruminal parakeratosis indicators. Further studies need to address if the ruminal microbiome was affected by the different treatments and if EPA was indeed successfully deposited in the lamb's meat and edible fats.

## Material and methods

### Animals, diets, and management

The experimentation involving live animals was conducted under strict compliance with international guidelines (Directive 2010/63/EU) regulating the use of production animals in animal experimentation, at the INIAV-Santarém facilities. The INIAV-Santarém facilities are certified by the competent veterinary authority (DGAV) to conduct animal experimentation (Ref: 04211000/000/2013). The experimental animal procedures were approved by the Ethical and Animal Well-Being Commission (CEBEA) of the Faculty of Veterinary Medicine, University of Lisbon, Portugal (Protocol FMV/CEBEA 007/2016). Animal management, handling, transport, and sacrifice were conducted replicating approved standard commercial practices regarding animal welfare, except that animals were individually housed. The study was also carried out in compliance with the ARRIVE guidelines.

Twenty-eight Merino Branco ram lambs were reared with dams on extensive grazing until weaning at approximately 60 days of age. After that, lambs were transported to INIAV—Santarém facilities, and randomly allocated to individual pens (1.52 m^2^) with wood shaving beds, and with free access to clean water. Animals were allocated to one of 4 groups of 7 lambs each, and randomly allocated to diets, following a completely randomized design. Lamb’s initial live weight averaged 21.8 ± 4.4 kg.

The C diet consisted of pellets containing dehydrated lucerne, barley and soybean meal (Table 4) and no added sources of EPA. The other diets maintained the same ingredients and proportion of C diet plus: SD diet—123 g/kg of spray-dried *N. oceanica* biomass; FD diet—92 g/kg freeze-dried *N. oceanica* biomass; O diet—12 g/kg of *Nannochloropsis* sp. free oil. The amounts of microalgae biomass or oil added to diets was determined to supply identical quantities of EPA (≈ 3 g/kg DM). The analysis of the final diets demonstrated that FD diet contained less EPA than the SD diet (Table 4), probably due to losses during handling, feed production, or differences among slurry batches. Diets containing *Nannochloropsis* were supplemented with 1.67 g/kg DM tocopheryl acetate (3a700 Vitamine E, 500 mg/g). Control diet was not supplemented because the premix already contained vitamin E in levels close to the NRC^47^ requirements for growing-finishing lambs of 20–30 kg of body weight. Diets were not formulated to be isoproteic or isocaloric, but crude protein ranged from 191 to 211 g/kg DM.

The *N. oceanica* biomasses were produced at Allmicroalgae industrial plant located in Pataias, Portugal. Cultures were autotrophically grown in Guillard's F2 medium as previously described^48^. Around three months after inoculation, the microalgal biomass was harvested from the photobioreactors, concentrated in a membrane system, subjected to a short-term high temperature treatment and dried in an industrial spray dryer to obtain the SD *N. oceanica*. The conditions were as follow: outlet temperature 85–90 °C and inlet temperature 200–210 °C. Frozen batches of slurry *N. oceanica* biomass were freeze-dried using a Scanvac Cool-safe Superior Touch freeze dryer (Scanvac, Denmark) with the following conditions: temperature, − 92 °C; initial ressure 0.2 mbar; final pressure 0.07 mbar. The *Nannochloropsis sp*. oil was purchased from Qualitas Health (Houston, TX, USA). Feed ingredients were mixed and pelleted (3 mm diameter) at Instituto Superior de Agronomia, Universidade de Lisboa experimental feed mill.

During the adaptation period of 8 days, lambs were dewormed against gastrointestinal and pulmonary nematodes by dosing with Sinvermin (Lapsa—Portuguesa Pecuária Lda., Portugal) and coccidiosis by dosing with Vecoxan (Elanco GmbH—Germany) and vaccinated against enterotoxaemia with Miloxan (Merial Labs., Spain). After that, they went through a transition period of 6 days where they were given1/3 of the pelleted experimental diet plus 2/3 of the basal ground feed ration, on the 1st and 2nd days and then the proportion of the experimental diet was regularly increased for the remaining days, until reaching 3/3. The experimental period started on the 14th day post-arrival when the diet was exclusively composed by the experimental pellets and lasted for 3 weeks. During the first two weeks of the experiment, 1.2 kg of feed was offered once a day (0900 am) and thereafter animals were fed ad libitum. Feed intake was measured during the entire experiment and averaged 1.19 ± 0.13 kg of DM/day.

### Slaughter procedures and sampling

At the end of the trial period, lambs were weighed at 0830 am at the housing facilities, without previous fasting, and immediately transported (circa 1 km) to the experimental abattoir of the INIAV—Santarém. Immediately after the slaughter, the whole rumen, abomasum, and lower intestine (cecum) digestive contents were collected from each lamb and frozen at − 80 °C. After that, they were freeze-dried, milled, and re-stored at − 80 °C until FA analysis. Faeces were collected directly from the rectum, frozen at − 80 °C, freeze-dried, milled and re-stored at − 80 °C. Samples of the ruminal wall (5 × 5 cm) were collected after washing the ruminal mucosa with tap water and fixed in 10% buffered formalin for histological examination. Ruminal content was straightened through 4 layers of cheesecloth and pH was measured in strained rumen fluid using a pH meter (Digital pH meter "pH-2005"; JP Selecta S.A, Barcelona, Spain) An aliquot of strained rumen fluid was immediately stored at − 20 °C for volatile fatty acids (VFA) analyses and another aliquot preserved with 2 mL of 10% formalin solution and stored at 2 °C until microscopic examination for rumen ciliate protozoa characterization.

### Rumen mucosa evaluation

Immediately after evisceration, the rumen was opened, emptied, and washed with tap water. Digital pictures (image size 16 mp) of macroscopic representative selected areas of the ruminal wall were taken with a Nikon D5100 Digital SLR camera (Nikon Europe BV, Badhoevedorp, The Netherlands) under constant lightning and photographic conditions. After, a 5 × 5 square from the ventral sac mucosa was collected for histopathological evaluation of parakeratotic lesions. Digital pictures were subjected to computational colorimetry tests on the mucosa colour (greyscale values) as an indicator of the degree of keratinization^46^, using image processing with a developed Python 3.8 script.

Samples from the ventral sac were fixed by immersion in 10% buffered formalin for at least 24 h. After fixation, samples were processed for paraffin embedding. Sections (3 μm thick) from each fragment were stained with haematoxylin and eosin for routine microscopic examination. From the 2 fragments corresponding to the ruminal wall of one animal, the 5 better preserved papillae were selected for histometry analysis, which was performed using a BX 511 microscope (Olympus, Tokyo, Japan) and the images were digitally captured using a DP 11 camera (Olympus, Tokyo, Japan) under a magnification of 20 × . The measurements were made using DP-Soft (Olympus) and ImageJ 1.43 softwares (ImageJ, Health National Institute of Mental, Bethesda).

### Rumen protozoa counting

Protozoal densities were obtained individually by microscopic counting as previously described^37^. Ciliate cell numbers were determined in duplicate for each sample and the identification at the *genus* level was made based on protozoa morphology, according to others^49^.

### Chemical analysis

Chemical analysis of diets was obtained as the average of the results of two pooled samples of each diet and analysed as previously described^50^. Acid-insoluble ash of feed and faeces, mainly consisting in silica, was determined gravimetrically after drying, ashing, boiling of ash in hydrochloric acid (HCl), filtering and washing of the hot hydrolysate, and re-ashing^51^. Ruminal volatile FA (VFA) were determined by gas chromatography with flame ionization detection (GC-FID) using a Shimadzu GC 2010-Plus (Shimadzu, Kyoto, Japan) equipped with a Nukol (30 m × 0.25 mm, 0.20 µm film thickness, Supelco, Bellefonte, PA, USA) capillary column and quantification was made using calibration curves according to others^52^.

Fatty acid methyl esters (FAME) of feed samples were prepared according to Sukhija and Palmquist^53^. Freeze-dried rumen, abomasum and lower intestine contents, and faeces samples were prepared by direct transesterification by reaction with sodium methoxide (0.5 M) in methanol at 50 °C for 15 min followed by addition of hydrogen chloride (1.25 M) in methanol at 80 °C for 20 min. Methyl nonadecanoate (1 mg/mL) was added as internal standard. Fatty acid methyl esters and DMA were analysed by GC-FID using a Shimadzu GC 2010-Plus (Shimadzu, Kyoto, Japan) equipped with an SP-2560 (100 m × 0.25 mm, 0.20 µm film thickness, Supelco, Bellefonte, PA, USA) capillary column. The chromatographic conditions were as follow: injector and detector temperatures were set at 250 °C and 280 °C, respectively; helium was used as the carrier gas at 1 mL/min at a constant flow; the initial oven temperature of 50 °C was held for 1 min, increased at 50 °C/min to 150 °C and held for 20 min, increased at 1 °C/min to 190 °C and then increased at 2 °C/min to 220 °C and held for 40 min. Identification of FAME and DMA was achieved by comparison of fatty acid retention times with those of commercial standards (FAME mix 37 components from Supelco Inc., Bellefont, PA, USA) and with published chromatograms^26^. Additional confirmation of FAME and DMA was achieved by electron impact mass spectrometry using a Shimadzu GC–MS QP2010 Plus (Shimadzu, Kyoto, Japan). The chromatographic column and the GC conditions were like the ones in the GC-FID analysis.

### Scanning electron microscopy (SEM) of *N. oceanica* biomass

Spray-dried and FD microalgae samples were mounted on aluminium stubs with carbon tape and coated with an 8 nm thick palladium-gold film in a Quorum Q150T ES sputtering system. *N. oceanica* surface morphology was observed in a Carl Zeiss AURIGA Crossbeam SEM–FIB workstation, using an accelerating voltage of 5 keV with an aperture size of 30 microns.

### Calculations and statistical analysis

The biohydrogenation estimates (disappearance, %) in the rumen for 18:2n-6, 18:3n-3, 20:4n-6 and 20:5n-3, were obtained using the diminishing abundance of these FA, proportional to the sum of C18 FA or of C20 FA, between diet and rumen, assuming that no losses of FA occur in the gastric compartments as shown below in the Eq. (1).

1 $${\mathrm{Biohydrogenation_{UFA} }}({\%}) =\frac{(\left[UFA-D\right]-\left[UFA-R\right])}{[UFA-D]}\times 100$$

where [UFA-D] and [UFA-R] are the proportions of each dietary unsaturated FA (UFA) expressed as % of total C18 FA or as % of total C20 FA, respectively for C18 UFA or C20 UFA.

C18 biohydrogenation completeness (%) was estimated considering the maximum 18:0 in the rumen and abomasal digesta, assuming a complete biohydrogenation of the C18 FA from the diet^12^. The calculations exemplified for rumen are presented in the Eq. (2):

2 $${\mathrm{C}18\, \mathrm{ biohydrogenation\,completeness }}\,({\%}) = \frac{[SA-R]}{[Max SA-R]}\times 100$$

The [SA-R] is the proportion of 18:0 in the rumen digesta and the [Max SA-R] is the maximum 18:0 proportion in the rumen, both expressed as % of total C18 FA, assuming that 100% of dietary unsaturated C18 FA biohydrogenated is converted to 18:0 and computed as shown in the Eq. (3).

3 $$\left[ {{\text{Max SA-R}}} \right] = \left( {\left[ {{\text{AO-D}}} \right] - \left[ {{\text{AO-R}}} \right]} \right) + \left( {\left[ {{\text{LA-D}} } \right] - \left[ {{\text{LA-R}} } \right]} \right) + \left( {\left[ {{\text{LnA-D}}} \right] - \left[ {{\text{LnA-R}} } \right]} \right) + \left[ {{\text{SA-D}} } \right]$$

Where: [SA-R], [AO-R], [LA-R], [LnA-R] are respectively the proportions of 18:0, *c*9-18:1, 18:2n-6 and 18:3n-3 in the rumen, and [SA-D], [AO-D], [LA-D], [LnA-D] the proportion of the same FA in the diets, expressed as % of total C18 FA.

The whole tract apparent digestibility (WTAD) of EPA, which included both biohydrogenation and post-ruminal digestion, was calculated using silica as the internal digestibility marker using marker and nutrients concentration ratios as described in Eq. (4):

4 $$WTAD\, \left(\%\right)=100- \left(100\times \left(\frac{{[Marker]}_{diet}}{{[Marker]}_{faeces}}/\frac{{[Nutrient]}_{faeces}}{{[Nutrient}_{]diet}}\right)\right)$$

Post ruminal digestibility (PRD) of EPA was estimated using both WTAD and biohydrogenation values, by computing the balance between the proportion of EPA escaping rumen metabolization (EPA_RE) and the proportion of EPA escaping the digestive tract as faecal excretion (EPA_FE). PRD calculations are presented in the Eq. (5).

5 $${{PRD }}\,({\%}) = \frac{\left(EPA\_RE\right)-(EPA\_FE)}{EPA\_RE}\times 100$$

where: EPA_RE (%) = 100—% EPA biohydrogenation, and EPA_FE (%) = 100—% EPA WTAD.

Chemical analysis (FAME and DMA), histometric data, ruminal pH and ruminal mucosa greyscale evaluation and FA data were analysed as a completely randomized experimental design using the MIXED procedure of SAS 9.4 (SAS Institute Inc., Cary, NC), using diet as a fixed factor and the animal as the experimental unit. When needed, the group option of the repeated statement was included in the model to accommodate the variance heterogeneity. Least square means (LSM) and standard error of the mean (SEM) are reported, and main effects and their interactions were considered significant at *P* < 0.05 and trends toward significance at 0.05 < *P* < 0.10. Due to an experimental incident the night before the slaughter, two animals receiving C diets accidentally had access to diets containing *Nannochloropsis* and consequently, those two animals were removed from the analysis.

## Supplementary Information

Supplementary Information.

## Data Availability

All data generated or analysed during this study are included in this published article (and its Supplementary Information files).
