# Supplementary material for: Freeze-dried Nannochloropsis oceanica biomass protects eicosapentaenoic acid (EPA) from metabolization in the rumen of lambs
Source: Sci Rep. 2021 Nov 8;11:21878. doi: 10.1038/s41598-021-01255-w (PMC8576006; doi:10.1038/s41598-021-01255-w)
Supplement: Supplementary file 1 — Supplementary Information. [file 41598_2021_1255_MOESM1_ESM.pdf]

Freeze-dried *Nannochloropsis oceanica* biomass protects eicosapentaenoic acid (EPA) from metabolization in the rumen of lambs

Ana C. M. Vítor[1,2], Alexandra E. Francisco[2,3], Joana Silva[4], Mário Pinho[1,2], Sharon A. Huws[5], José Santos-Silva[2,3], Rui J.B. Bessa[1,2], Susana P. Alves[1,2*]

[1] Faculdade de Medicina Veterinária, Universidade de Lisboa, Av. da Universidade Técnica, 1300-477, Lisboa, Portugal

[2] CIISA - Centro de Investigação Interdisciplinar em Sanidade Animal, Avenida da Universidade Técnica, 1300-477 Lisboa, Portugal

[3] Polo de Investigação de Santarém, Instituto Nacional de Investigação Agrária e Veterinária (INIAV-Santarém), 2005-048 Vale de Santarém, Portugal

[4] Allmicroalgae, Rua 25 Abril, 2445-413 Pataias, Portugal

[5] School of Biological Sciences, Institute for Global Food Security, Queen's University Belfast, Belfast, United Kingdom

Supplementary material are presented below

Table S1. Fatty acid (FA) and dimethyl acetal (DMA) composition in the rumen (% of identified peaks).

Item	Diets[1]				*P*-value
	C	O	SD	FD	
**Fatty acids**					
12:0	0.27 ± 0.030	0.26 ± 0.025	0.30 ± 0.059	0.34 ± 0.019	0.061
i-13:0	0.07^{c} ± 0.008	0.14^{b} ± 0.009	0.21^{a} ± 0.011	0.23^{a} ± 0.006	<0.001
13:0	0.07 ± 0.016	0.07 ± 0.010	0.08 ± 0.013	0.08 ± 0.014	0.970
i-14:0	0.45 ± 0.042	0.42 ± 0.056	0.51 ± 0.047	0.66 ± 0.068	0.064
14:0	0.97^{c} ± 0.273	1.20^{c} ± 0.110	1.78^{b} ± 0.092	2.53^{a} ± 0.117	<0.001
i-15:0	0.45^{ab} ± 0.059	0.48^{a} ± 0.050	0.58^{a} ± 0.075	0.34^{b} ± 0.031	0.017
a-15:0	2.00^{ab} ± 0.412	2.01^{a} ± 0.175	1.34^{b} ± 0.100	1.20^{b} ± 0.136	0.005
15:0	1.25 ± 0.167	1.09 ± 0.097	1.23 ± 0.125	1.02 ± 0.076	0.398
16:0	17.14^{c} ± 0.700	22.20^{b} ± 0.897	28.41^{a} ± 0.560	28.37^{a} ± 0.599	<0.001
i-16:0	0.60 ± 0.141	0.35^{a} ± 0.057	0.27 ± 0.029	0.33 ± 0.111	0.137
*c*9-16:1	0.12^{c} ± 0.023	1.57^{b} ± 0.186	2.36^{b} ± 0.607	5.58^{a} ± 0.453	<0.001
t-16:1	0.11^{b} ± 0.024	0.22^{ab} ± 0.099	0.38^{a} ± 0.058	0.35^{a} ± 0.058	<0.001
i-17:0	0.12 ± 0.016	0.16 ± 0.031	0.16 ± 0.027	0.11 ± 0.035	0.406
a-17:0	0.42^{a} ± 0.113	0.33^{ab} ± 0.074	0.18^{b} ± 0.009	0.21^{b} ± 0.015	0.034
17:0	0.65 ± 0.084	0.64 ± 0.062	0.65 ± 0.068	0.56 ± 0.042	0.538
17:0-cyclo[2]	0.31^{a} ± 0.070	0.29^{a} ± 0.053	0.15^{b} ± 0.026	0.15^{b} ± 0.034	0.041
17:1	0.12 ± 0.023	0.12 ± 0.029	0.06 ± 0.016	0.08 ± 0.009	0.160
18:0	26.39^{a} ± 4.473	10.94^{bc} ± 0.876	16.91^{ab} ± 3.237	9.09^{c} ± 0.821	0.002
*t*6/*t*7/*t*8-18:1	0.88 ± 0.207	1.18 ± 0.194	0.96 ± 0.171	1.04 ± 0.077	0.745
*t*9-18:1	0.41 ± 0.141	0.75 ± 0.126	0.77 ± 0.124	0.74 ± 0.047	0.185
*t*10-18:1	14.0^{a} ± 4.236	10.50^{a} ± 3.246	3.22^{b} ± 1.022	5.49^{ab} ± 1.209	0.032
*t*11-18:1	2.31^{b} ± 0.715	6.24^{ab} ± 1.858	7.38^{a} ± 1.356	6.51^{a} ± 1.369	0.006
*t*12-18:1	0.75 ± 0.079	0.66 ± 0.138	1.04 ± 0.105	0.92 ± 0.137	0.104
*t*13/*t*14/*c*9-18:1	7.23 ± 0.870	6.42 ± 0.462	5.73 ± 0.842	6.42 ± 0.450	0.680
*t*15-18:1	0.56^{a} ± 0.066	0.24^{b} ± 0.086	0.59^{a} ± 0.086	0.43^{ab} ± 0.079	0.028
*c*11-18:1	0.987 ± 0.156	1.06 ± 0.134	0.94 ± 0.080	1.15 ± 0.108	0.345
*c*12-18:1	0.35 ± 0.065	0.27 ± 0.065	0.53 ± 0.098	0.36 ± 0.028	0.202
*c*13-18:1	0.05 ± 0.010	0.05 ± 0.008	0.07 ± 0.008	0.06 ± 0.011	0.315
*t*16/*c*14-18:1	0.34 ± 0.062	0.15 ± 0.074	0.38 ± 0.077	0.23 ± 0.064	0.118
*c*15-18:1	0.27^{ab} ± 0.075	0.29^{a} ± 0.061	0.12^{b} ± 0.020	0.25^{ab} ± 0.074	0.019
*c*16-18:1	0.08 ± 0.009	0.11 ± 0.013	0.09 ± 0.017	0.11 ± 0.016	0.404
t,*t*-18:2	0.09 ± 0.023	0.17 ± 0.048	0.05 ± 0.009	0.06 ± 0.012	0.059
*t*9,*c*12-18:2	0.07^{b} ± 0.012	0.16^{ab} ± 0.054	0.14^{ab} ± 0.051	0.21^{a} ± 0.049	0.037
*t*11,*c*15/*t*10,*c*15-18:2	0.45^{bc} ± 0.105	1.49^{a} ± 0.359	0.26^{c} ± 0.069	0.65^{b} ± 0.150	0.006
18:2n-6	9.027 ± 1.148	7.99 ± 0.680	4.94 ± 1.147	6.95 ± 0.863	0.083
*c*9,*t*11-CLA	n.d.	0.10 ± 0.011	0.19 ± 0.053	0.12 ± 0.026	0.228
t,*t*-CLA	0.17^{a} ± 0.010	0.11^{b} ± 0.011	0.18^{ab} ± 0.033	0.32^{a}b ± 0.117	0.002
18:3n-3	1.69 ± 0.148	1.81 ± 0.127	1.78 ± 0.056	1.57 ± 0.072	0.138

oxo-18:0	0.64 ± 0.239	1.58 ± 0.395	1.55 ± 0.275	1.21 ± 0.290	0.077
20:0	0.60 ± 0.048	0.50 ± 0.038	1.49 ± 0.481	0.61 ± 0.117	0.104
20:2n-6	0.12 ± 0.064	0.29 ± 0.052	0.20 ± 0.053	0.27 ± 0.042	0.174
20:3n-6	n.d.	0.24 ± 0.066	0.17 ± 0.044	0.27 ± 0.024	0.101
20:4n-6	0.07^{d} ± 0.041	0.29^{c} ± 0.057	0.76^{b} ± 0.106	1.62^{a} ± 0.122	<0.001
20:4n-3	n.d.	0.83 ± 0.311	0.27 ± 0.103	0.38 ± 0.075	0.232
20:5n-3	n.d.	1.14^{b} ± 0.185	1.75^{b} ± 0.327	3.47^{a} ± 0.356	<0.001
21:0	0.06^{b} ± 0.004	0.14^{a} ± 0.025	0.17^{a} ± 0.037	0.15^{a} ± 0.034	0.001
22:0	0.56^{a} ± 0.025	0.54^{ab} ± 0.062	0.47^{b} ± 0.027	0.43^{b} ± 0.020	0.005
23:0	0.26 ± 0.048	0.18 ± 0.019	0.19 ± 0.026	0.17 ± 0.014	0.309
24:0	0.55^{a} ± 0.026	0.46^{ab} ± 0.041	0.41^{bc} ± 0.018	0.37^{c} ± 0.006	<0.001
24:1	0.09^{a} ± 0.008	0.07^{ab} ± 0.009	0.08^{ab} ± 0.015	0.06^{b} ± 0.007	0.024
26:0	0.27^{a} ± 0.024	0.23^{ab} ± 0.024	0.19^{b} ± 0.004	0.18^{b} ± 0.014	0.017
28:0	0.20^{a} ± 0.010	0.15^{b} ± 0.009	0.18^{ab} ± 0.014	0.11^{c} ± 0.007	<0.001
**DMA**					
i-14:0	0.11^{b} ± 0.028	0.18^{b} ± 0.045	0.30^{a} ± 0.033	0.20^{ab} ± 0.050	0.003
14:0	0.12^{c} ± 0.040	0.22^{bc} ± 0.048	0.45^{a} ± 0.063	0.34^{ab} ± 0.057	0.001
i-15:0	0.05 ± 0.007	0.08 ± 0.025	0.10 ± 0.014	0.06 ± 0.012	0.086
a-15:0	0.31^{b} ± 0.053	0.41^{ab} ± 0.056	0.56^{a} ± 0.046	0.33^{b} ± 0.069	0.009
15:0	0.31^{a} ± 0.060	0.22^{ab} ± 0.025	0.18^{bc} ± 0.018	0.14^{c} ± 0.017	0.016
i-16:0	0.71 ± 0.256	0.37 ± 0.089	0.38 ± 0.083	0.49 ± 0.083	0.498
16:0	2.353^{a} ± 0.224	2.05^{a} ± 0.086	1.36^{b} ± 0.104	1.50^{b} ± 0.172	<0.001
18:0	0.17^{a} ± 0.026	0.08^{b} ± 0.014	0.08^{b} ± 0.016	0.06^{b} ± 0.017	0.020
t-18:1	0.46^{a} ± 0.062	0.26^{b} ± 0.043	0.23^{b} ± 0.028	0.21^{b} ± 0.018	0.008
*c*9-18:1	0.109 ± 0.005	0.14 ± 0.045	0.19 ± 0.047	0.15 ± 0.035	0.080

Values are means ± standard error of the mean. Means within a row with different letters are significantly different ($P < 0.05$). [1]Diets (or treatments): C, control; O, Control plus 1.2% *Nannochloropsis* sp. oil; SD, control plus 12.3% spray-dried *Nannochloropsis oceanica*; FD, control plus 9.2% freeze-dried *N. oceanica*. [2]17:0-cyclo-17, 11-cyclohexyl-11:0.

Table S2. Fatty acid and dimethyl acetal composition in the abomasum (% of identified peaks).

Item	Diets[1]				*P*-value
	C	O	SD	FD	
**Fatty acids**					
12:0	$0.25^{b} \pm 0.025$	$0.34^{ab} \pm 0.050$	$0.33^{ab} \pm 0.074$	$0.38^{a} \pm 0.032$	0.028
13:0	$0.13^{b} \pm 0.014$	$0.19^{ab} \pm 0.030$	$0.20^{ab} \pm 0.034$	$0.24^{a} \pm 0.021$	0.002
i-14:0	$0.12^{b} \pm 0.016$	$0.21^{ab} \pm 0.050$	$0.24^{a} \pm 0.040$	$0.35^{a} \pm 0.048$	<0.001
14:0	$0.82^{d} \pm 0.216$	$1.35^{c} \pm 0.094$	$1.68^{b} \pm 0.091$	$2.52^{a} \pm 0.103$	<0.001
15:0	1.05 ± 0.167	1.23 ± 0.136	1.02 ± 0.182	1.05 ± 0.074	0.673
i-15:0	$0.38^{b} \pm 0.029$	$0.50^{ab} \pm 0.052$	$0.58^{a} \pm 0.039$	$0.39^{b} \pm 0.043$	0.004
a-15:0	$2.219^{a} \pm 0.128$	$1.98^{a} \pm 0.229$	$1.38^{b} \pm 0.090$	$1.20^{b} \pm 0.148$	<0.001
16:0	$17.33^{c} \pm 0.341$	$22.20^{b} \pm 1.095$	$27.43^{a} \pm 0.408$	$28.21^{a} \pm 0.584$	<0.001
i-16:0	0.57 ± 0.119	0.42 ± 0.084	0.29 ± 0.038	0.38 ± 0.105	0.117
*c*9-16:1	$0.15^{c} \pm 0.018$	$1.90^{b} \pm 0.278$	$2.42^{b} \pm 0.365$	$5.60^{a} \pm 0.414$	<0.001
16:0-Me_4	$0.10^{b} \pm 0.021$	$0.23^{ab} \pm 0.091$	$0.42^{a} \pm 0.058$	$0.25^{ab} \pm 0.077$	<0.001
17:0	0.877 ± 0.061	0.84 ± 0.082	0.74 ± 0.050	0.70 ± 0.063	0.4559
i-17:0	$0.18^{c} \pm 0.027$	$0.29^{abc} \pm 0.067$	$0.45^{a} \pm 0.046$	$0.32^{b} \pm 0.034$	<0.001
a-17:0	$0.43^{a} \pm 0.105$	$0.38^{ab} \pm 0.093$	$0.20^{b} \pm 0.012$	$0.11^{c} \pm 0.031$	0.005
*c*9-17:1	$0.16^{ab} \pm 0.028$	$0.19^{a} \pm 0.024$	$0.09^{b} \pm 0.019$	$0.18^{a} \pm 0.015$	0.006
17:0-cyclo[2]	$0.31^{ab} \pm 0.088$	$0.36^{a} \pm 0.072$	$0.20^{bc} \pm 0.028$	$0.15^{c} \pm 0.033$	0.058
18:0	$28.30^{a} \pm 6.451$	$12.92^{b} \pm 1.212$	$16.93^{b} \pm 2.937$	$9.47^{c} \pm 0.610$	0.003
oxo-18:0	$0.48^{b} \pm 0.170$	$1.27^{a} \pm 0.281$	$1.48^{a} \pm 0.202$	$1.03^{a} \pm 0.282$	0.007
*t*6/*t*7/*t*8-18:1	0.93 ± 0.219	1.25 ± 0.219	1.16 ± 0.145	1.09 ± 0.067	0.755
*t*9-18:1	0.656 ± 0.135	0.74 ± 0.165	0.89 ± 0.116	0.78 ± 0.056	0.347
*t*10-18:1	$13.69^{ab} \pm 3.855$	$13.08^{a} \pm 3.307$	$3.69^{c} \pm 1.084$	$5.56^{bc} \pm 1.219$	0.018
*t*11-18:1	$2.31^{b} \pm 0.734$	$7.43^{ab} \pm 2.560$	$8.87^{a} \pm 2.139$	$6.96^{a} \pm 1.648$	0.008
*t*12-18:1	$0.78^{b} \pm 0.090$	$0.88^{ab} \pm 0.228$	$1.26^{a} \pm 0.082$	$1.00^{ab} \pm 0.156$	0.006
*t*13/*t*14/*c*9-18:1	$7.83^{a} \pm 0.767$	$6.99^{a} \pm 0.308$	$5.45^{b} \pm 0.373$	$6.88^{a} \pm 0.355$	0.010
*t*15-18:1	0.55 ± 1.110	0.46 ± 0.185	0.69 ± 0.092	0.39 ± 0.083	0.159
*c*11-18:1	$0.84^{b} \pm 0.093$	$1.02^{ab} \pm 0.201$	$1.02^{ab} \pm 0.090$	$1.28^{a} \pm 0.104$	0.040
*c*12-18:1	0.41 ± 0.059	0.38 ± 0.067	0.72 ± 0.136	0.44 ± 0.026	0.176
*c*13-18:1	0.09 ± 0.017	0.08 ± 0.023	0.11 ± 0.006	0.09 ± 0.016	0.327
*t*16/*c*14-18:1	0.43 ± 0.093	0.21 ± 0.088	0.47 ± 0.080	0.28 ± 0.059	0.110
*c*15-18:1	$0.30^{a} \pm 0.054$	$0.38^{a} \pm 0.053$	$0.19^{b} \pm 0.018$	$0.27^{ab} \pm 0.066$	0.012
*c*16-18:1	0.11 ± 0.018	0.15 ± 0.013	0.16 ± 0.016	0.15 ± 0.021	0.304
*t*9,*c*12-18:2	$0.08^{b} \pm 0.012$	$0.22^{a} \pm 0.041$	$0.22^{a} \pm 0.035$	$0.25^{a} \pm 0.038$	<0.001
*t*11,*c*15/*t*10,*c*15-18:2	$0.49^{bc} \pm 0.131$	$1.89^{a} \pm 0.430$	$0.33^{c} \pm 0.064$	$0.76^{b} \pm 0.182$	0.005
18:2n-6	$9.86^{a} \pm 1.447$	$7.96^{ab} \pm 1.020$	$3.90^{c} \pm 0.424$	$6.60^{b} \pm 0.702$	<0.001
18:3n-3/20:1	$0.51^{ab} \pm 0.285$	$0.92^{ab} \pm 0.313$	$1.08^{a} \pm 0.114$	$0.27^{b} \pm 0.078$	0.003
*c*9,*t*11-CLA	n.d.	$0.13^{b} \pm 0.030$	$0.26^{a} \pm 0.033$	$0.17^{ab} \pm 0.031$	0.034
20:0	0.64 ± 0.050	0.64 ± 0.066	1.30 ± 0.364	0.60 ± 0.048	0.312
20:2n-6	$0.14^{b} \pm 0.052$	$0.49^{a} \pm 0.090$	$0.52^{a} \pm 0.107$	$0.47^{a} \pm 0.101$	0.002
20:3n-6	$0.04^{b} \pm 0.218$	$0.68^{a} \pm 0.078$	$0.49^{ab} \pm 0.092$	$0.45^{ab} \pm 0.076$	0.044

20:4n-6	0.07^{d} ± 0.014	0.36^{c} ± 0.049	0.79^{b} ± 0.064	1.62^{a} ± 0.116	<0.001
20:5n-3	n.d.	1.44^{b} ± 0.192	1.60^{b} ± 0.151	3.38^{a} ± 0.390	0.001
21:0	0.02 ± 0.071	0.12 ± 0.031	0.15 ± 0.025	0.14 ± 0.030	0.426
22:0	0.56^{b} ± 0.037	0.80^{a} ± 0.048	0.54^{b} ± 0.045	0.53^{b} ± 0.030	0.001
24:0	0.61^{a} ± 0.035	0.56^{a} ± 0.036	0.43^{b} ± 0.011	0.38^{c} ± 0.016	<0.001
26:0	0.28^{a} ± 0.011	0.30^{a} ± 0.024	0.22^{b} ± 0.013	0.19^{b} ± 0.018	<0.001
**DMA**					
i-14:0	0.10^{b} ± 0.017	0.15^{ab} ± 0.027	0.24^{a} ± 0.050	0.19^{ab} ± 0.052	0.041
14:0	0.12^{c} ± 0.035	0.20^{bc} ± 0.038	0.35^{a} ± 0.044	0.32^{ab} ± 0.044	0.001
a-15:0	0.14 ± 0.045	0.17 ± 0.067	0.06 ± 0.010	0.24 ± 0.154	0.102
15:0	0.27^{a} ± 0.028	0.21^{ab} ± 0.024	0.16b ± 0.022	0.16^{b} ± 0.037	0.021
16:0	1.63^{a} ± 0.183	1.31^{ab} ± 0.114	0.83^{c} ± 0.050	0.99^{bc} ± 0.134	<0.001
18:1	0.41 ± 0.074	0.30 ± 0.067	0.27 ± 0.036	0.24 ± 0.024	0.178

Values are means ± standard error of the mean. Means within a row with different letters are significantly different (P < 0.05). [1]Diets (or treatments): C, control; O, Control plus 1.2% *Nannochloropsis* sp. oil; SD, control plus 12.3% spray-dried *Nannochloropsis oceanica*; FD, control plus 9.2% freeze-dried *N. oceanica*. [2]17:0-cyclo-17, 11-cyclohexyl-11:0.

Table S3. Fatty acid and dimethyl acetal composition in the cecum (% of identified peaks).

Item	Diets[1]				*P*-value
	C	O	SD	FD	
**Fatty acids**					
12:0	0.42 ± 0.079	0.43 ± 0.120	0.60 ± 0.116	0.58 ± 0.038	0.210
13:0	0.11[b] ± 0.016	0.18[b] ± 0.046	0.37[a] ± 0.051	0.54[a] ± 0.123	<0.001
i-14:0	0.56 ± 0.071	0.69 ± 0.115	1.10 ± 0.315	0.84 ± 0.091	0.089
14:0	1.24[c] ± 0.166	1.69[c] ± 0.329	2.93[b] ± 0.364	3.99[a] ± 0.298	<0.001
i-15:0	0.53 ± 0.075	0.82 ± 0.227	0.89 ± 0.123	0.70 ± 0.121	0.104
a-15:0	2.577 ± 0.489	2.69 ± 0.394	2.31 ± 0.358	2.24 ± 0.121	0.656
15:0	1.88 ± 0.184	1.74 ± 0.211	2.43 ± 0.280	2.10 ± 0.143	0.222
i-16:0	1.06 ± 0.231	0.60 ± 0.057	0.86 ± 0.175	0.81 ± 0.154	0.126
16:0	20.26[d] ± 0.274	23.79[c] ± 0.866	28.75[b] ± 0.334	31.44[a] ± 0.559	<0.001
t-16:1	0.07[b] ± 0.014	0.12[ab] ± 0.035	0.21[a] ± 0.027	0.21[a] ± 0.037	0.001
*c*7-16:1	0.08[b] ± 0.016	0.20[a] ± 0.034	0.16[a] ± 0.034	0.24[a] ± 0.036	0.001
*c*9-16:1	0.09[d] ± 0.026	0.88[c] ± 0.115	2.24[b] ± 0.320	3.99[a] ± 0.514	<0.001
i-17:0	0.64[c] ± 0.060	0.74[bc] ± 0.126	1.38[a] ± 0.205	0.92[ab] ± 0.100	0.008
a-17:0	0.99 ± 0.240	0.67 ± 0.086	0.97 ± 0.153	0.59 ± 0.076	0.109
17:0	2.15 ± 0.220	1.80 ± 0.230	2.15 ± 0.300	1.97 ± 0.177	0.683
17:1	0.17 ± 0.046	0.16 ± 0.025	0.22 ± 0.086	0.18 ± 0.038	0.919
17:0-cyclo[2]	0.44 ± 0.119	0.48 ± 0.123	0.31 ± 0.086	0.35 ± 0.096	0.658
18:0	28.92[a] ± 5.692	14.37[b] ± 2.678	13.77[b] ± 3.483	10.77[b] ± 1.071	0.026
i-18:0	0.29 ± 0.058	0.18 ± 0.063	0.40 ± 0.173	0.22 ± 0.040	0.433
*t*6/*t*7/*t*8-18:1	0.74 ± 0.209	0.54 ± 0.195	0.58 ± 0.149	0.51 ± 0.080	0.765
*t*9-18:1	0.37 ± 0.098	0.63 ± 0.173	0.40 ± 0.109	0.36 ± 0.040	0.515
*t*10-18:1	9.53[a] ± 2.955	8.35[ab] ± 3.041	1.92[b] ± 0.881	2.41[b] ± 0.803	0.042
*t*11-18:1	3.08 ± 0.812	7.24 ± 2.975	4.57 ± 0.905	3.83 ± 0.564	0.437
*t*12-18:1	0.69 ± 0.078	0.52 ± 0.038	0.70 ± 0.132	0.49 ± 0.063	0.148
*t*15-18:1	0.45[a] ± 0.054	0.14[c] ± 0.020	0.28[b] ± 0.063	0.11[c] ± 0.026	<0.001
*t*16/*c*14-18:1	0.31 ± 0.074	0.11 ± 0.038	0.28 ± 0.067	0.14 ± 0.031	0.054
*c*9/*t*13/*t*14-18:1	6.13[ab] ± 0.869	5.63[a] ± 0.491	4.27[bc] ± 0.359	3.99[c] ± 0.215	0.012
*c*11-18:1	0.76 ± 0.101	0.99 ± 0.090	0.82 ± 0.089	0.87 ± 0.081	0.351
*c*12-18:1	0.57 ± 0.130	0.68 ± 0.190	0.70 ± 0.137	0.54 ± 0.061	0.671
*c*13-18:1	0.04 ± 0.004	0.05 ± 0.005	0.10 ± 0.021	0.06 ± 0.017	0.121
*c*15-18:1	0.22[b] ± 0.048	0.44[a] ± 0.079	0.13[b] ± 0.021	0.24[ab] ± 0.085	0.009
*c*16-18:1	0.13 ± 0.017	0.14 ± 0.032	0.08 ± 0.012	0.08 ± 0.017	0.062
t,*t*-18:2	0.08 ± 0.013	0.13 ± 0.030	0.10 ± 0.012	0.06 ± 0.007	0.053
t,*c*-18:2	0.07[b] ± 0.017	0.13[a] ± 0.015	0.14[a] ± 0.026	0.11[ab] ± 0.013	0.047
*t*11,*c*15/*t*10,*c*15-18:2	0.38[b] ± 0.114	1.14[a] ± 0.224	0.26[b] ± 0.053	0.39[b] ± 0.078	0.008
18:2n-6	3.28 ± 0.598	4.13 ± 0.531	2.81 ± 0.372	2.80 ± 0.300	0.179
18:3n-3/20:1	0.75[b] ± 0.104	1.16[a] ± 0.089	1.06[ab] ± 0.149	0.85[b] ± 0.052	0.018
*c*9,*t*11-CLA	0.311[b] ± 0.008	0.18[a] ± 0.015	0.30[ab] ± 0.128	0.23[a] ± 0.044	0.001
c,*t*-CLA	0.04 ± 0.026	0.08 ± 0.023	0.14 ± 0.038	0.24 ± 0.192	0.219

t,t-CLA	0.70 ± 0.118	1.48 ± 1.032	0.74 ± 0.284	1.87 ± 0.426	0.090
oxo-18:0	0.16 ± 0.062	0.32 ± 0.059	0.31 ± 0.066	0.21 ± 0.049	0.240
20:0	0.78 ± 0.053	0.79 ± 0.092	1.44 ± 0.403	0.80 ± 0.031	0.467
20:2n-6	0.39 ± 0.175	0.33 ± 0.061	0.52 ± 0.165	0.53 ± 0.104	0.386
20:3n-3	n.d.	0.67 ± 0.113	0.44± 0.103	0.60 ± 0.075	0.317
20:3n-6	0.05^{b} ± 0.004	0.19^{a} ± 0.047	0.28^{a} ± 0.049	0.19^{a} ± 0.034	<0.001
20:4n-6	0.19^{b} ± 0.052	0.40^{b} ± 0.106	1.55^{a} ± 0.255	1.92^{a} ± 0.181	<0.001
20:5n-3	n.d.	1.11^{b} ± 0.246	3.13^{a} ± 0.498	3.41^{a} ± 0.391	<0.001
21:0	0.08^{b} ± 0.006	0.12^{a} ± 0.012	0.18^{a} ± 0.035	0.14^{a} ± 0.017	0.002
22:0	0.79 ± 0.067	0.87 ± 0.079	0.85 ± 0.088	0.73 ± 0.029	0.231
23:0	0.31^{b} ± 0.026	0.39^{ab} ± 0.032	0.45^{a} ± 0.048	0.42^{a} ± 0.037	0.032
24:0	0.95 ± 0.062	1.00 ± 0.158	1.35 ± 0.214	1.07 ± 0.077	0.281
26:0	0.55 ± 0.051	0.72 ± 0.179	0.68 ± 0.066	0.65 ± 0.069	0.376
28:0	0.43 ± 0.034	0.59 ± 0.183	0.54 ± 0.086	0.50 ± 0.059	0.441
**DMA**					
i-140	0.12^{b} ± 0.021	0.24^{ab} ± 0.065	0.40^{a} ± 0.108	0.32^{a} ± 0.076	0.009
14:0	0.11^{b} ± 0.015	0.33^{ab} ± 0.140	0.72^{a} ± 0.161	0.66^{a} ± 0.127	<0.001
a-15:0	0.34^{b} ± 0.039	0.59^{ab} ± 0.164	0.63^{a} ± 0.065	0.53^{ab} ± 0.088	0.004
15:0	0.35 ± 0.132	0.28 ± 0.081	0.29 ± 0.062	0.26 ± 0.051	0.930
16:0	2.43 ± 0.844	2.77 ± 0.799	2.39 ± 0.409	2.51 ± 0.291	0.978
18:0	0.31 ± 0.093	0.23 ± 0.040	0.34 ± 0.092	0.25 ± 0.036	0.652
18:1	0.61 ± 0.257	0.47 ± 0.157	0.55 ± 0.158	0.47 ± 0.053	0.913

Values are means ± standard error of the mean. Means within a row with different letters are significantly different ($P < 0.05$). [1]Diets (or treatments): C, control; O, Control plus 1.2% *Nannochloropsis* sp. oil; SD, control plus 12.3% spray-dried *Nannochloropsis oceanica*; FD, control plus 9.2% freeze-dried *N. oceanica*. [2]17:0-cyclo-17, 11-cyclohexyl-11:0.
